# Community structured model for vaccine strategies to control COVID19 spread: A mathematical study

**DOI:** 10.1371/journal.pone.0258648

**Published:** 2022-10-27

**Authors:** Elena Aruffo, Pei Yuan, Yi Tan, Evgenia Gatov, Effie Gournis, Sarah Collier, Nick Ogden, Jacques Bélair, Huaiping Zhu

**Affiliations:** 1 Centre for Diseases Modeling (CDM), York University, Toronto, Ontario, Canada; 2 Department of Mathematics and Statistics, York University, Toronto, Ontario, Canada; 3 Toronto Public Health, Toronto, Ontario, Canada; 4 Public Health Agency of Canada, Ottawa, Ontario, Canada; 5 Département de Mathématiques et de Statistique, Université de Montréal, Montréal, Québec, Canada; Nanyang Technological University, SINGAPORE

## Abstract

Initial efforts to mitigate the COVID-19 pandemic have relied heavily on non-pharmaceutical interventions (*NPIs*), including physical distancing, hand hygiene, and mask-wearing. However, an effective vaccine is essential to containing the spread of the virus. We developed a compartmental model to examine different vaccine strategies for controlling the spread of COVID-19. Our framework accounts for testing rates, test-turnaround times, and vaccination waning immunity. Using reported case data from the city of Toronto, Canada between Mar-Dec, 2020 we defined epidemic phases of infection using contact rates as well as the probability of transmission upon contact. We investigated the impact of vaccine distribution by comparing different permutations of waning immunity, vaccine coverage and efficacy throughout various stages of NPI’s relaxation in terms of cases and deaths. The basic reproduction number is also studied. We observed that widespread vaccine coverage substantially reduced the number of cases and deaths. Under phases with high transmission, an early or late reopening will result in new resurgence of the infection, even with the highest coverage. On the other hand, under phases with lower transmission, 60% of coverage is enough to prevent new infections. Our analysis of *R*_0_ showed that the basic reproduction number is reduced by decreasing the tests turnaround time and transmission in the household. While we found that household transmission can decrease following the introduction of a vaccine, public health efforts to reduce test turnaround times remain important for virus containment.

## Introduction

Since the World Health Organization declared the rapid spread of SARS-CoV-2 a global pandemic on March 11, 2020 [1], initial control measures that are still in use in most affected countries are non-pharmaceutical interventions (*NPIs*, including physical distancing, hand hygiene, and mask-wearing), which have been efficient in controlling virus spread [2–4]. However, due to SARS-CoV-2 rapid spread and high mortality, it is widely accepted that a safe and effective vaccine is crucial for mitigating the epidemic [5, 6]. The scientific community has immediately started studying the virus and developing a vaccine against it. Several such vaccines are already available to multiple countries [7, 8], with high efficacy against the virus [9]. Given limited available doses and the increasing epidemic trend, a safe and effective vaccination strategy is paramount. Planning for the optimal distribution of the vaccine, and ultimately, determining whether and at what point the safe relaxation of NPIs is possible are critical public health objectives [5, 6].

Several studies have quantified the effect of COVID-19 vaccination by using mathematical models [10–17]. For example, one study has utilized an information-dependent SIRI model and considered the occurrence of re-infection [14], showing that case counts can be reduced when vaccination is introduced into the population. However, latent stage, mortality, and implementation of quarantine/self-isolation were not considered. Another study has incorporated vaccination and isolation as key parameters in a SEIR model and compared the results of different vaccination coverage and isolation days [15]. While this study found that long isolation and high vaccine coverage can decrease virus spread, it did not account for hospitalizations, different vaccine efficacy levels, the degree of waning immunity, and the presence of asymptomatic infections, which are known to play an important role in spreading the virus [18, 19]. Since NPIs have been the first response to control the spread of the infection, many studies show the impact that the combination of relaxing NPIs and vaccine have on the infections [20–23]. These works confirm that lifting NPIs after introducing vaccination is not an efficient strategy.

Many questions remain about the duration of immunity and some models were developed to address the impact that waning immunity might have on possible resurgence of COVID-19 [24–26]. Although these studies present important results for public health and infection control, the specific context around vaccine implementation has not been investigated in depth. Many regions have escalated/de-escalated public health measures in timed stages to control virus spread [27–29]. The implementation of these policy phases depends on the local epidemic conditions and test processes. Accordingly, vaccine implementation should also consider the stages of the local epidemic trajectory. Lastly, despite the importance of detection of positive cases and test turnaround times [30], to our knowledge, few studies have considered testing processes in the context of vaccine modeling.

To fill these gaps, we aimed to develop an extensive compartmental model to investigate the impact of different scenarios of vaccine implementation on COVID-19 cases and deaths and the basic reproductive number. We simulate different degrees of waning immunity, vaccine efficacy, and population coverage, along with different time points at which NPIs are relaxed, in order to determine, under different conditions, the vaccine coverage needed to prevent a possible COVID-19 resurgence. To make our work more widely applicable to different regions, we consider different phases of the local epidemic trajectory and related public health mitigation strategies, as well as testing processes (including turnaround time).

## Materials and methods

### Community structured transmission model

The model we are proposing is an extension of the Susceptible-Infectious-Recovered framework, where demographics are ignored and death due to infection is included. The infectious compartment is divided into latent and non-infectious period (E), infectious period, where individuals will or will not develop symptoms (*I*_*a*_, *I*), isolation period (*I*_*q*_) and hospitalization period (*H*). The recovered compartment is divided into full recovery from infection or death. Herein, we will refer to our model as *SEI*_*a*_*II*_*q*_*HDR*. We defined different phases using public health data related to policy (i.e., lockdowns, re-opening), test positivity, and test-turnaround times. To better reflect individuals’ activities as they relate to these phases, we divided the daily routine of the population into hours spent at home, in the community, or at work/school, each partition is represented by its own probability of transmission term and average contact rate. We introduced a variable G which is set as 1 if hours are spent in the household and 0 when the hours are spent outside. The vaccination process is included in the model by reducing the susceptible class and moving immunized individuals into the recovered compartment.

COVID-19 transmission occurs as follows: susceptible individuals become infected after encountering infectious asymptomatic and symptomatic cases, at rate *βc*. However, since symptomatic cases are assumed to stay at home, they contribute only to household transmission. After the latent period, a proportion *b* shows symptoms, while 1−*b* is asymptomatic. The latter is assumed to recover at rate $\gamma_{I_{a}}$. A proportion *ρ* of symptomatic individuals get tested, and if positive, isolated. Once tested, a proportion (*r*_*q*24_) will receive results within 24 hours at rate *γ*_24_ and the rest will receive results within 48 hours, at rate *γ*_48_. Isolated cases are assumed to have no contact; hence they do not transmit the infection. Symptomatic and isolated cases might develop severe symptoms (at rate *γ*_*mh*_, *γ*_*qh*_) and become hospitalized, or recover from mild status (at rate *γ*_*mr*_, *γ*_*qr*_). We assumed that only hospitalized patients might die from infection, at rate *μ*_*H*_. Once the vaccine is introduced into the population, immunity through inoculation might wane over time, making individuals susceptible again, at rate *ω*.

The vaccination process is informed using a piecewise function defined as follows:

$$f(t)=\left\{ \begin{array}{c} 0,if\;0\leq t<T_{0}\\ \phi t-\phi T_{0}\;if\;T_{0}\leq t<T_{0}+T_{1}\\ -\phi t+\phi (T_{0}+2T_{1}),\;if\;T_{0}+T_{1}\leq t<{2T}_{1}\\ 0,\;if\;t\geq 2T_{1} \end{array}\right.$$ (1)

Where *T*_0_ indicates the time at which vaccination starts, *T*_1_ is the time at which peak of the function is reached *T*_*v*_ = 2*T*_1_ is the time needed to complete vaccination process, and $\int_{T_{0}}^{T_{v}}f(t)=1$, then we can calculate *ϕ* = 1/*T*_1_ which is the peak value of the function.

Data show that the daily vaccine doses follow a bell curve shape [31] and, although other functions can represent this shape, the chosen function *f*(*t*) is the most convenient.

**Fig 1:**
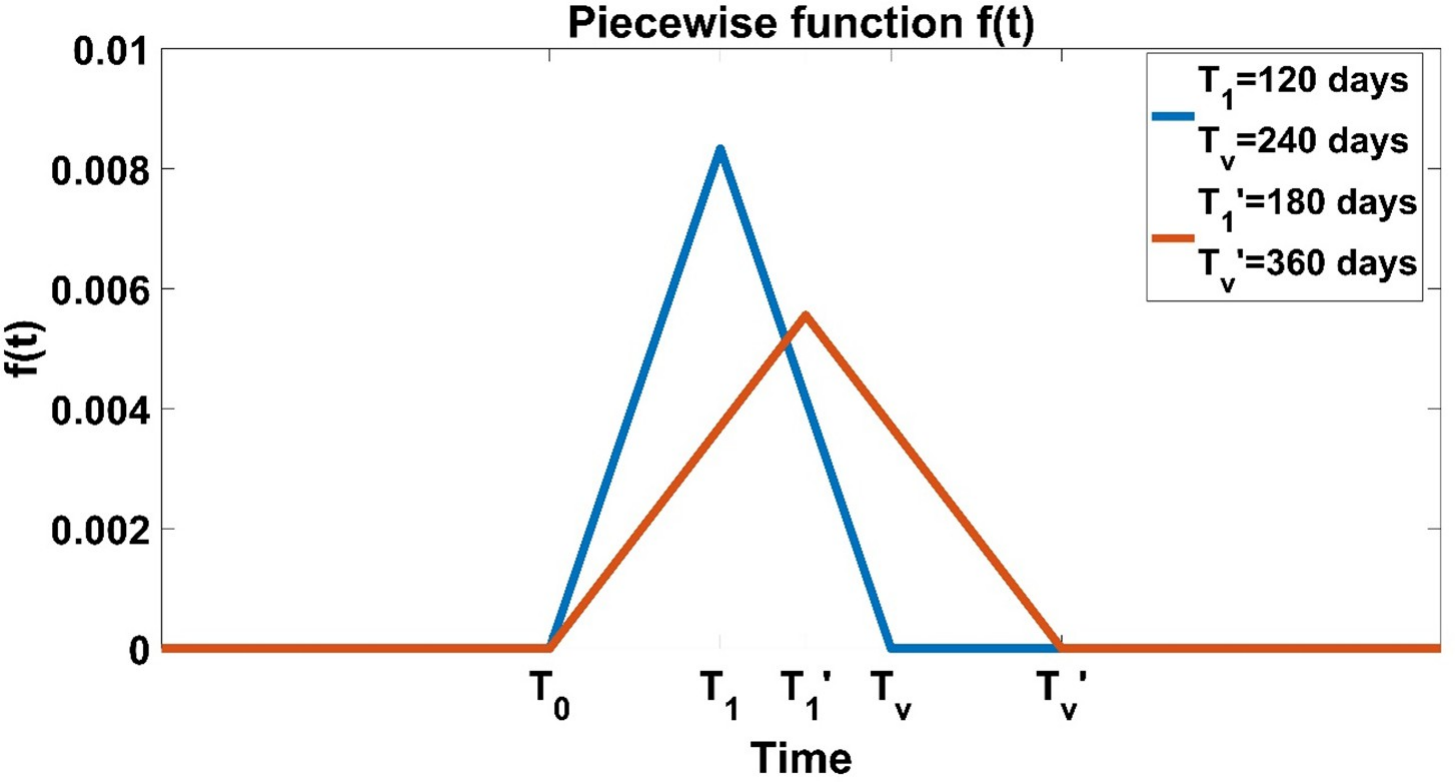
Example of the piecewise function f(t) in Eq (1). Here, we present the shape of the function when the vaccination process is completed after 240 days (blue) or 360 days (orange) after the beginning of vaccination (*T*_0_).

Fig 1 provides an example of the function describing the vaccination process. We observe how the shape of the function changes when the entire time needed to vaccinate the population changes.

**Fig 2:**
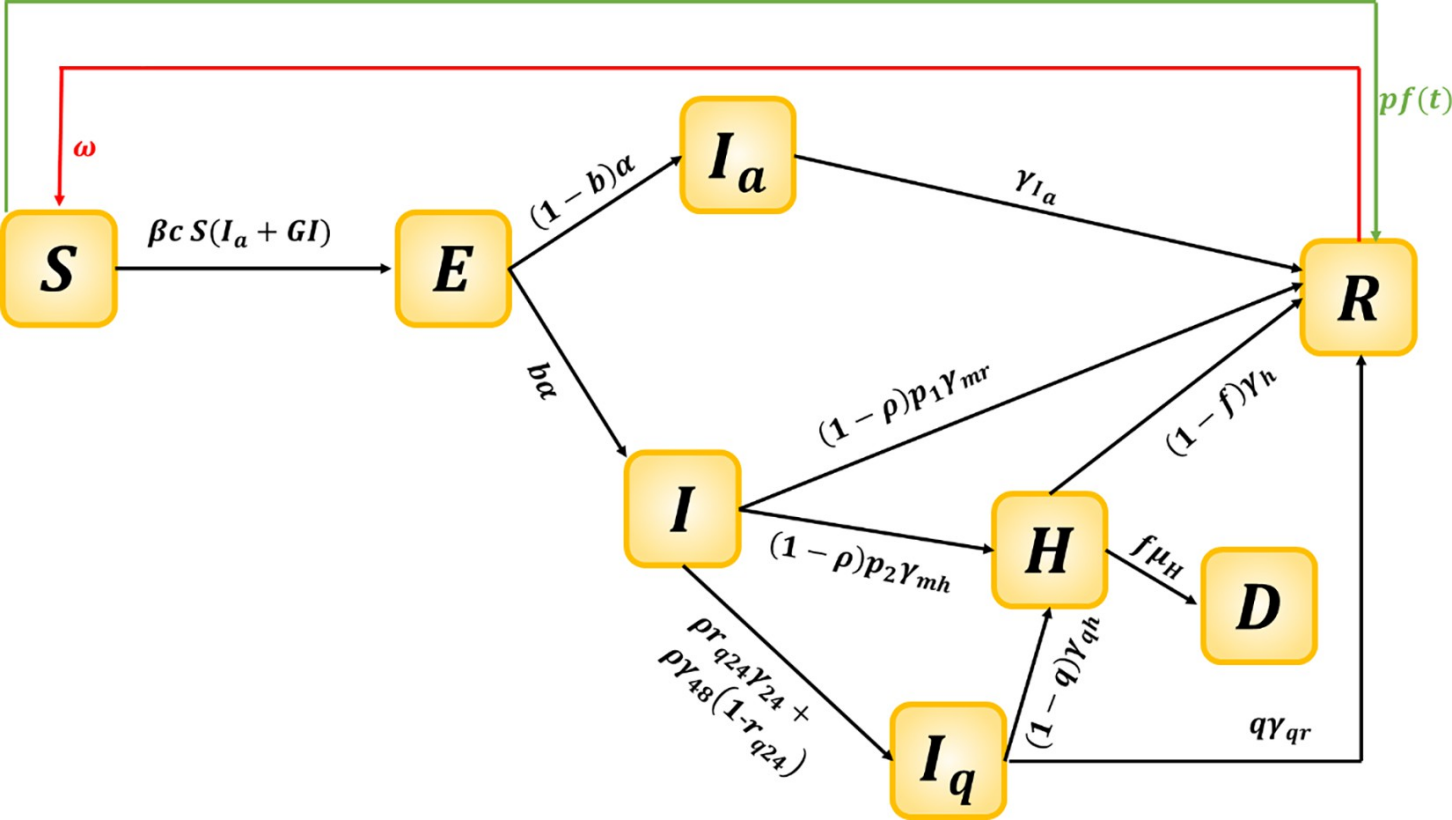
Flow diagram of model (2). The vaccine process is defined with green line, while the waning immunity process is indicated with red line.

The general system of ODEs describing COVID-19 infection dynamics is given by (**1**).

$$\begin{array}{l} S^{\prime }=-p\epsilon f(t)S_{T0}-\beta_{lk}c_{lk}S(I_{a}+{GI}_{m})+\omega R\\ E^{\prime }=\beta_{lk}c_{lk}S(I_{A}+{GI}_{m})-\alpha E\\ I_{a}^{\prime }=(1-b)\alpha E-\gamma_{Ia}I_{a}\\ I\prime =b\alpha E-(1-\rho )(p_{1_{k}}\gamma_{mr}+p_{2_{k}}\gamma_{mh})I-\rho r_{q24_{k}}\gamma_{24}I-\rho \gamma_{48}{(1-r}_{q24_{k}})I\\ I_{q}^{\prime }=\rho r_{q24_{k}}\gamma_{24}I_{m}+\rho \gamma_{48}{(1-r}_{q24_{k}})I-{(1-q_{k})\gamma }_{qh}I_{q}-q_{k}\gamma_{qr}I_{q}\\ H^{\prime }=(1-\rho )p_{2_{k}}\gamma_{mh}I+{(1-q_{k})\gamma }_{qh}I_{q}-{(1-f_{k})\gamma }_{H}H-f_{k}\mu_{H}H\\ D^{\prime }=f_{k}\mu_{H}H\\ R^{\prime }=p\epsilon f(t)S_{T0}+(1-\rho )p_{1_{k}}\gamma_{mr}I+q_{k}\gamma_{qr}I_{q}+(1-f_{k})\gamma_{H}H+\gamma_{Ia}I_{a}-\omega R \end{array}$$ (2)

where *l* indicates the different locations (*l* = *c*,*h*) and *k* indicated the different phases, *p* is the proportion of the population that needs to be vaccinated, $\frac{1}{\omega }$ as the waning period, *ϵ* is the vaccine efficacy and *S*_*T*0_ is the susceptible population at time *T*_0_. The dynamic flow diagram is shown in Fig 2. The list of the parameters and assumptions are provided in Tables 1 and 2, respectively.

**Table 1:** Model parameters.

Parameter	Definition	Value	Ref
*β*_*hk*_	Probability of transmission in the house (*k* = 1,2,3,4 indicating the phases of the infection)	6.72e-08	Estimated
		2.65e-08	
		6.86.e-08	
		5.8e-08	
*β*_*ck*_	Probability of transmission in the community (*k* = 1,2,3,4 indicating the phases of the infection)	3.5e-09	Estimated
		2.5e-09	
		2.99e-09	
		3e-09	
*c*_*h*_	Average daily contact at home	2.4	[34]
${c_{c}}_{k}$	Average daily contact in the community (*k* = 1,2,3,4 indicating the phases of the infection)	${c_{c}}_{1}$ = 4.378646	Estimated
		${c_{c}}_{2}=7.421244$	
		${c_{c}}_{3}$ = 7.905791	
		${c_{c}}_{4}$ = 6.423287	
${c_{w}}_{k}$	Average daily contact in the community/work (*k* = 1,2,3,4 indicating the phases of the infection)	${c_{w}}_{1}=6.839068$	Estimated
		${c_{w}}_{2}$ = 8.914671	
		${c_{w}}_{3}$ = 9.93748	
		${c_{w}}_{4}$ = 9.709616	
*α*	Average latent period	¼ *days*^−1^	[35, 36]
*b*	Proportion of symptomatic cases	0.8	[37]
*ρ*_*k*_	Testing proportion (*k* = 1,2,3,4 indicating the phases of the infection)	*ρ*_1,2,3,4_ = 0.635	[38]
${p_{1}}_{k}$	Proportion of mild cases not tested who will recover as mild (*k* = 1,2,3,4 indicating the phases of the infection)	${p_{1}}_{k}=1-{p_{2}}_{k}$	Calculated
${p_{2}}_{k}$	Proportion of mild cases not tested who will become hospitalized (*k* = 1,2,3,4 indicating the phases of the infection)	${p_{2}}_{1}$ = 0.144	Calculated
		${p_{2}}_{2}=0.0663$	
		${p_{2}}_{3}=0.0469$	
		${p_{2}}_{4}=0.0794$	
$\gamma_{I_{a}}$	Recovery rate of mild cases, not tested	0.07 *days*^−1^	[39]
*γ*_*mr*_	Recovery rate of mild cases, not tested	1/14 *days*^−1^	[40]
*γ*_*qr*_	Recovery rate of quarantined cases	1/14 *days*^−1^	Assumed as *γ*_*mr*_
*γ*_*mh*_	Hospitalization rate of non-tested mild cases	1/6 *days*^−1^	[41], assumed
1/*γ*_24,28_	Time needed to return tests	24 hours, 48 hours	Assumed
$r_{q24_{k}}$	Proportion of individuals receiving their test result within 24 hours (*k* = 1,2,3,4 indicating the phases of the infection)	$r_{q24_{1}}$ = 0.5	[33], assumed
		$r_{q24_{2}}$ = 0.45;	
		$r_{q24_{3}}$ = 0.37;	
		$r_{q24_{4}}$ = 0.37;	
*γ*_*qh*_	Hospitalization rate of quarantined cases	${\gamma_{qh}}_{1}$ = 0.121469	Estimated
		${\gamma_{qh}}_{2}$ = 0.028572	
		${\gamma_{qh}}_{3}$ = 0.091	
		${\gamma_{qh}}_{4}$ = 0.05552	
*γ*_*H*_	Recovery rate of hospitalized	1/10 *days*^−1^	[41], assumed
*q*_*k*_	Proportion of mild cases recovered (*k* = 1,2,3,4 indicating the phases of the infection)	$q_{k}={p_{1}}_{k}$	Assumed
1−*q*_*k*_	Proportion of mild cases recovered (*k* = 1,2,3,4 indicating the phases of the infection)		Calculated
*f*_*k*_	Proportion of hospitalized cases deceased (*k* = 1,2,3,4 indicating the phases of the infection)	*f*_1_ = 0.6601	Calculated
		*f*_2_ = 0.6985	
		*f*_3_ = 0.2261	
		*f*_4_ = 0.7075	
1−*f*_*k*_	Proportion of hospitalized cases recovered (*k* = 1,2,3,4 indicating the phases of the infection)		Calculated
*ω*	Waning periods	No waning, 3, 6, 12 months	Assumed
*p*	Vaccination coverage	10%, 30%, 60%, 90%	Assumed
*ϵ*	Vaccine efficacy	70%, 90%	Assumed
${\mu_{H}}_{k}$	Mortality rate (*k* = 1,2,3,4 indicating the phases of the infection)	$\mu_{H_{1}}=0.208928$	Estimated
		${\mu_{H}}_{2}$ = 0.026316	
		${\mu_{H}}_{3}$ = 0.12499	
		${\mu_{H}}_{4}$ = 0.05552	
*G*	Switch parameter (G=1 home hours- G=0 community hours)	G=1 home hours	Assumed
		G=0 community hours	
*E*_0_	Initial values of exposed individuals	270	Estimated
*A*_0_	Initial values of asymptomatic individuals	29	Estimated
${I_{q}}_{0}$	Initial values of quarantined individuals	34	Estimated
${I_{m}}_{0}$	Initial values of mild individuals	112	Estimated
*H*_*o*_	Initial values of hospitalized cases	37	[33]
*D*_0_	Initial values deceased cases	0	[33]
*S*_0_	Initial values susceptible individuals	$2956024-E_{0}-A_{0}-{I_{m}}_{0}-{I_{q}}_{0}-R_{0}-H_{0}-D_{0}$	Calculated from [42]

**Table 2:** Model variables and assumptions.

Variable	Definition
*S*	Ssceptible individuals
*E*	Exposed (non-infectious) individuals
*I*_*a*_	Infectious asymptomatic individuals
*I*	Infectious symptomatic individuals with mild symptoms
*I*_*q*_	Infectious symptomatic isolated individuals with mild symptoms
*H*	Individuals needing extra health care
*D*	Deceased individuals from infection
*R*	Recovered individuals
**Model’s assumptions**	
Vaccination	Vaccination is introduced in the population starting December 15, 2020. We consider 10%, 30%, 60%, 90% vaccination coverage of the population. Once vaccinated, individuals are moved to the recovered compartment.
Waning immunity	We assume that from the recovered compartment, individuals will wane their immunity over 0, 90, 180 or 365 days.
Vaccine efficacy	We investigate different vaccine efficacies, 70% and 90%.
NPI’s relaxation	We assume that NPI’s are lifted either on May 1, 2021 or on December 1, 2021 (roughly after five or 12 months from the beginning of vaccine distribution). We assume that in this scenario the transmission and the average number of contacts in the households, workplace and community are the highest among all our estimates.
Phases and hours	We assume that the average time spend in the household and in the community depend on the phases. In particular, for phase 1,3, and 4 individuals spend two hours in the community, while phase 2 four hours.
Hospital	In our model the hospital compartment indicates any location where patients need extra health care, such as hospitals, long-term care, nursing homes.
Deceased cases	Only individuals from the hospital compartment can move to the fatal compartment.
Test turnaround time	We assume that all the tests are returned within 48 hours.
Isolated and mild cases	We assume that only infectious cases participate to the infection and they are assumed to stay at home once the symptoms appear. Isolated cases are assumed not to participate to the transmission.
Testing process	We assume that only a proportion *ρ* of symptomatic cases decide to get tested.

### Data source and phases

**Fig 3:**
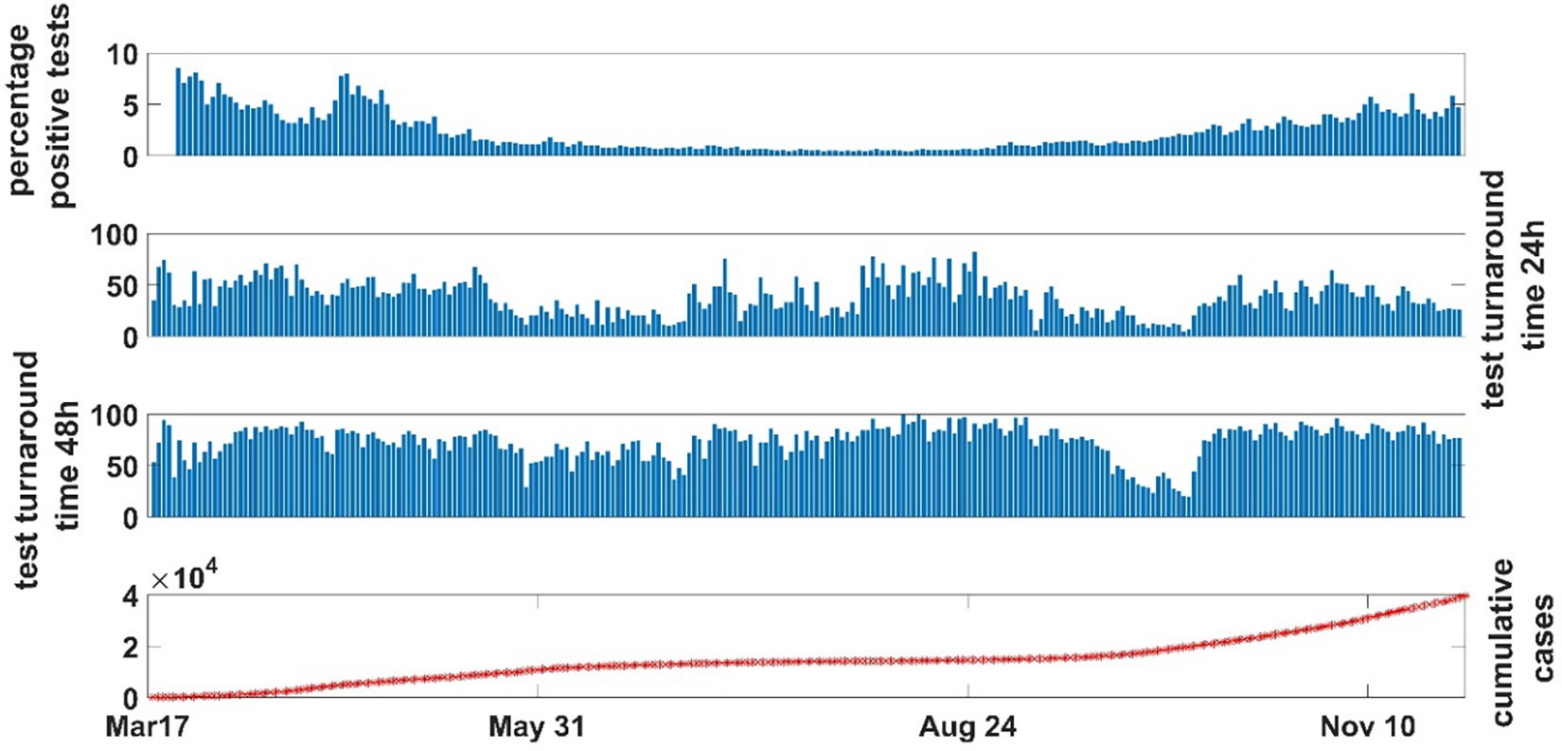
Percentage positive tests, tests turnaround times (24h-48h) and cumulative cases are reported from March 17, 2020 to November 28, 2020 in Toronto. The percentage positive tests decrease from May 31 to August 24, when it starts increasing, showing, however a magnitude smaller than the first wave in March. Until May 31 about 50% of the tests were returned within 24h, while until the end of August, the trend shows an initial decreasing followed by an increasing trend. After this, the curve decreases again, raising up in October and remaining below 50%. However, it slightly decreases again after November 10. Similar pattern for 48h tests turnaround time. The cumulative cases increase until May 31, and then remain stable until the end of August when the increase starts again and continue until Dec 2, 2020, with a slight decrease after the beginning of November.

We developed a novel modeling framework that can be easily applied to any geographical context. As a case study, we used publicly available data on reported cases and deaths, test turnaround times in Toronto from March 17 to December 2, 2020, and percentage positivity test in Ontario, Canada [32, 33] from April 19 17 to December 2, 2020, as well as information about the local epidemic trajectory and corresponding policy [33] around NPIs, school/business closures, testing processes and test turnaround times to define defined the following phases (Fig 3):

1. PHASE1, March 17 (April 19)-May 31 (Lockdown): Schools and public spaces are closed, resulting in limited time and contact in the community, with somewhat lenient NPIs. Testing is not widespread, with 50% of results returned within 24 hours and percent positivity between 8-10%. There was a slight increase in cumulative cases.
2. PHASE2, June 1-August 24 (Re-opening stages): Businesses gradually open, resulting in increased contact and time spent in the community, and stricter NPIs. Widespread testing is in place, with 24h turnaround under 50% initially, then rising in the summer months. Percent positivity dropped to 1.2%. Infections seem to have stabilized.
3. PHASE3, August 25-November 10 (Resurgence starts): Schools fully reopened in September, followed by escalating restrictions in October and November (e.g., closure of indoor dining, restriction on the number of people indoors), resulting in decreasing time in the community under strict NPIs. Testing is widespread, with less than 50% returned within 24 hours, and a 5% percent positivity. Case counts begin to rise more steeply, compared to before the March lockdown.
4. PHASE4, November 10-December 2 (Additional closures): Schools are open, with additional closures of public spaces. The public is encouraged to stay at home. Test turnaround within 24h is < 50%, with a 5% percent positivity. The sharp rise in cases stabilizes slightly.

Using the least square method, we fit our model to estimate parameters for each phase (see Table 1).

### Impact of vaccine introduction on COVID-19 trajectory

To study the impact of a vaccine and its biological processes on case numbers and deaths under different phases of the infection and different conditions, we provide a comprehensive analysis varying all these components. We compare different vaccine coverage (10%, 30%, 60%, 90%), assuming that the entire vaccination process is completed in 120 or 360 days. Given the vaccine efficacy uncertainty, we propose projections when this factor is 70% or 90%. Also, we investigate the effect of waning immunity comparing the scenarios in which waning immunity never occurs or occurs over three or 12 months. These scenarios are simulated in conjunction with NPIs relaxation, which occurs after five or 12 months from the beginning of vaccination process.

We investigate the basic reproduction number, with no waning immunity, by introducing four-dimensional contour plots. These plots allow us to investigate how the reproduction number is changing when three parameters are varied over given intervals and immunity does not wane. This provides a comprehensive understanding of the relationship between *R*_0_ and the parameters in our analysis. We examined the basic reproduction number *R*_0_ using the next-generation matrix method (details in Supporting Information). We derived the following expression:

$$R_{0}={R_{0}}_{I_{A}}+R_{0_{I}}$$ (3)

$${R_{0}}_{I_{a}}=\frac{(1-b)\beta cS_{0}}{\gamma_{Ia}}$$ (4)

$$R_{0_{I}}=\frac{Gb\beta cS_{0}}{\left(1-\rho )(p_{1}\gamma_{mr}+p_{2}\gamma_{mh})+\rho r_{q24}\gamma_{24}+\rho \gamma_{48}{(1-r}_{q24}\right)}$$ (5)

Eq (3) shows the contribution that asymptomatic and symptomatic cases have on generating new infections. Since symptomatic cases are always at home, this expression highlights the contribution that the household transmission has on the spread of the virus.

### Sensitivity analysis

To identify how significant the testing related parameters (*ρ*, *r*_*q*24_), time to reach the peak of the vaccination function (*time*_*v*_), proportion of vaccinated individuals (*p*) and waning rate (*ω*) are on changing the model outcomes, we conducted the sensitivity analysis on these parameters. We used Latin Hypercube Sampling/Partial Rank Correlation Coefficient (***LHS***/***PRCC***) [43]. We generated 1000 samples using a uniform distribution and evaluated the PRCC for the parameters sampled. A parameter is considered significant if the PRCC value is in magnitude larger than 0.5. Ranges of the values are reported in the (**S1 Table**). We conducted one sensitivity analysis using all the parameters mentioned, and one removing the waning rate.

## Results

### Reproduction number

**Fig 4:**
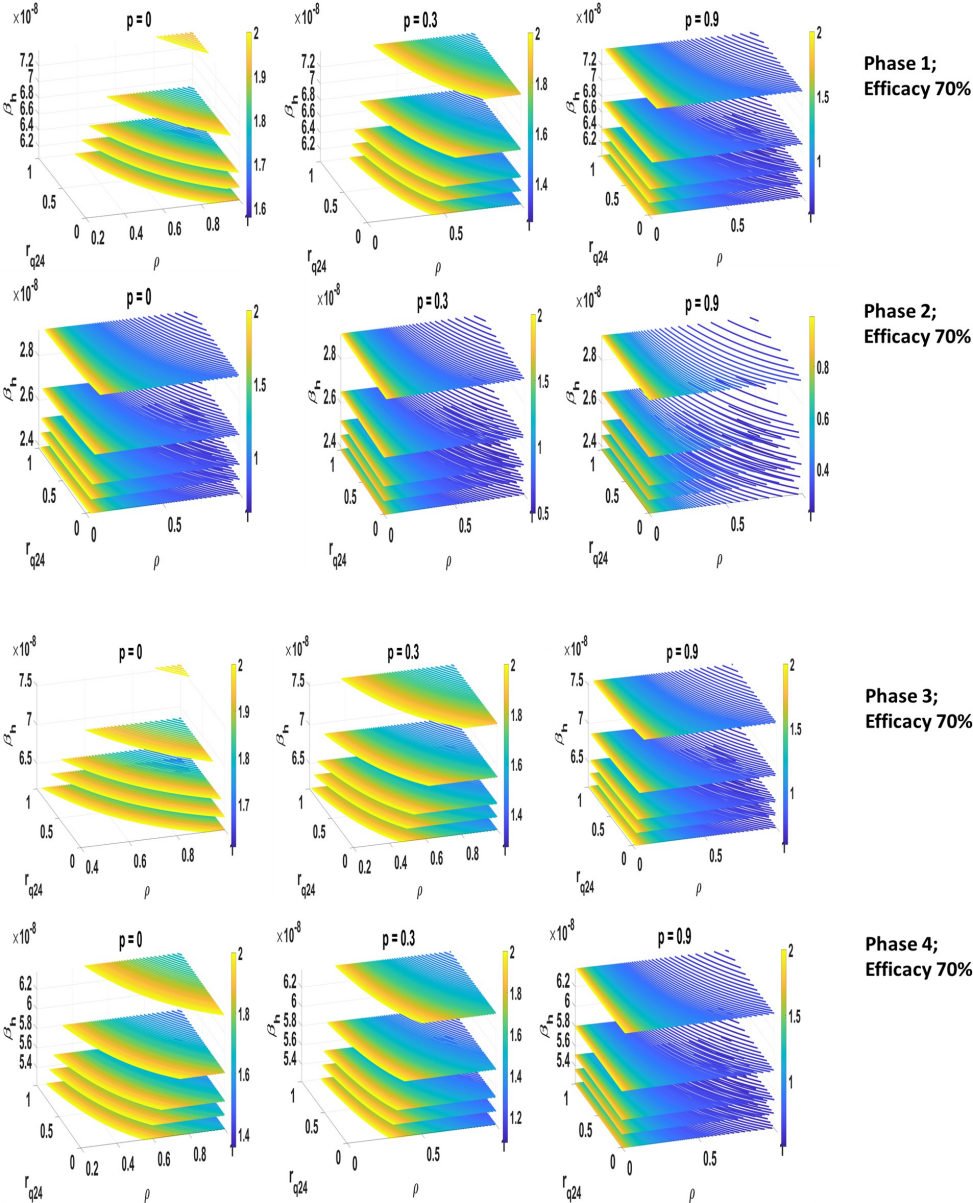
*R*_0_ 4D contour plot in the *β*_*h*_, *r*_24_, *ρ* parameters space. The values of the reproduction number are presented under Phase 1-2 and 3-4 (a, b, respectively) considering 0%, 30%,90% vaccine coverage, lifelong immunity and efficacy 70%. In all cases, we observe that as the vaccine coverage increases, the reproduction number decreases visibly. Also, the yellow region (indicating the highest values of the reproduction number) increases as the probability of the transmission in the household increases. In the phases with higher transmission (1, 3 and 4), we observe that the reproduction number is always greater than 1, except for 90% coverage. We also observe that if more people receive their test back within 24 hours, the reproduction number decreases. Similarly, in general, to maintain the reproduction number below 2, more than 20% of the infectious individuals need to be tested.

Fig 4 illustrates 4D contour plots of the household reproduction number in the *β*_*h*_−*r*_*q*24_−*ρ* parametric space. We present the scenario with no vaccination and with 30%, 90% vaccine coverage, 70% efficacy and lifelong immunity. As expected, the *R*_0_ decreases as the vaccination coverage increases and the probability of transmission decreases. With coverage of 90% it is possible to detect parametric regions in which the reproduction number is less than 1 in all the phases. Only with Phase 2 we can find parameters providing *R*_0_<1 for smaller coverages. In the scenarios with high transmission and contacts and small coverages (see Phase 1, 3 and 4), to maintain the reproduction number below 2, it is necessary to have either most of the symptomatic individuals tested or reducing the turnaround time. However, in general the proportion of symptomatic individuals getting tested (*ρ*) appears to be significant in reducing the spread of the infection. For example, in Phase 2, with a coverage of 0%-30%, *ρ* should be at least 20% to drop the reproduction number below 1; while in Phase 3, the coverage should be 90% and *ρ* above 70%. If the vaccine efficacy increases to 90% (see S1 Fig), the reproduction number becomes smaller even with scenarios of higher transmission, and the parametric region providing *R*_0_<1 is much larger. For example, in Phase 1 and Phase 3, a higher efficacy results in reducing the maximum value of the reproduction number by roughly 50% and 40%, respectively, with 90% coverage.

### Long term projections: cases and deaths

In this section, we report the dynamic of the infection if immunity does not wane or wanes 90, 180 and 365 days. Here, we present the scenarios with no waning or 180 days. We present scenarios whereby a 70% effective vaccine, is introduced in the population on December 15, 2020 (coverage: 10%, blue, 30%, red, 60% yellow, 90% purple). Scenarios with 90% efficacy is reported in Supporting Information. NPI’s relaxed on May 1, 2021 or on December 1, 2021 (roughly after five or 12 months after the vaccine distribution starts). We also report predictions when the vaccination process is completed in 120 or 360 days, defined as fast and slow rollout, respectively. All the other scenarios are in the Supporting Information.

### Predictions under Phase 1, 3 and 4

**Fig 5:**
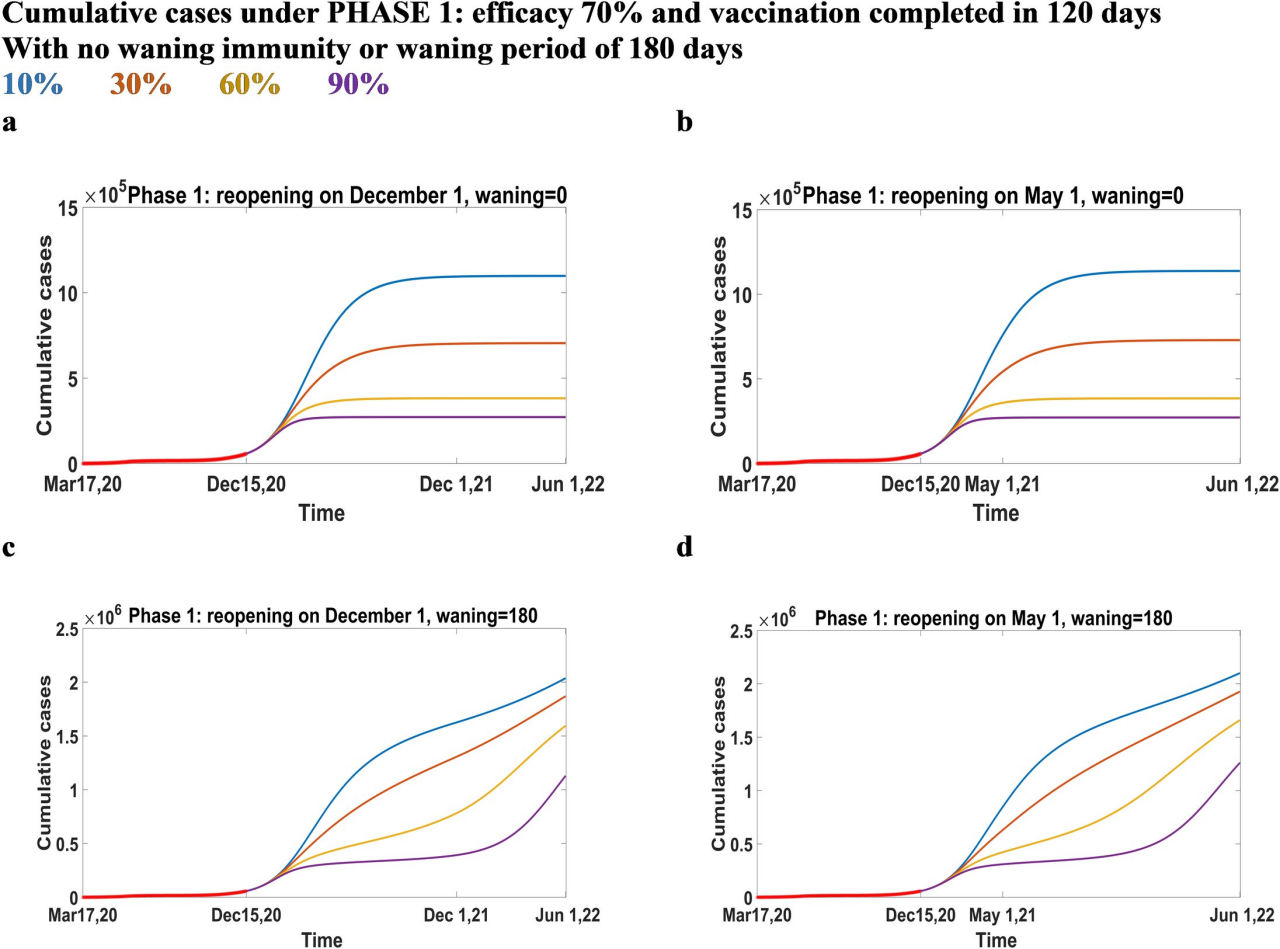
Cumulative cases under Phase 1: Efficacy 70% and vaccination completed in 120 days. Cumulative cases when the vaccine efficacy is 70%, vaccination is completed in 120 days under Phase 1 conditions, reopening in December 2021 (a-c) or May 2021 (b-d) and immunity does not wane (a-b) or wanes over 180 days (c-d).

**Fig 6:**
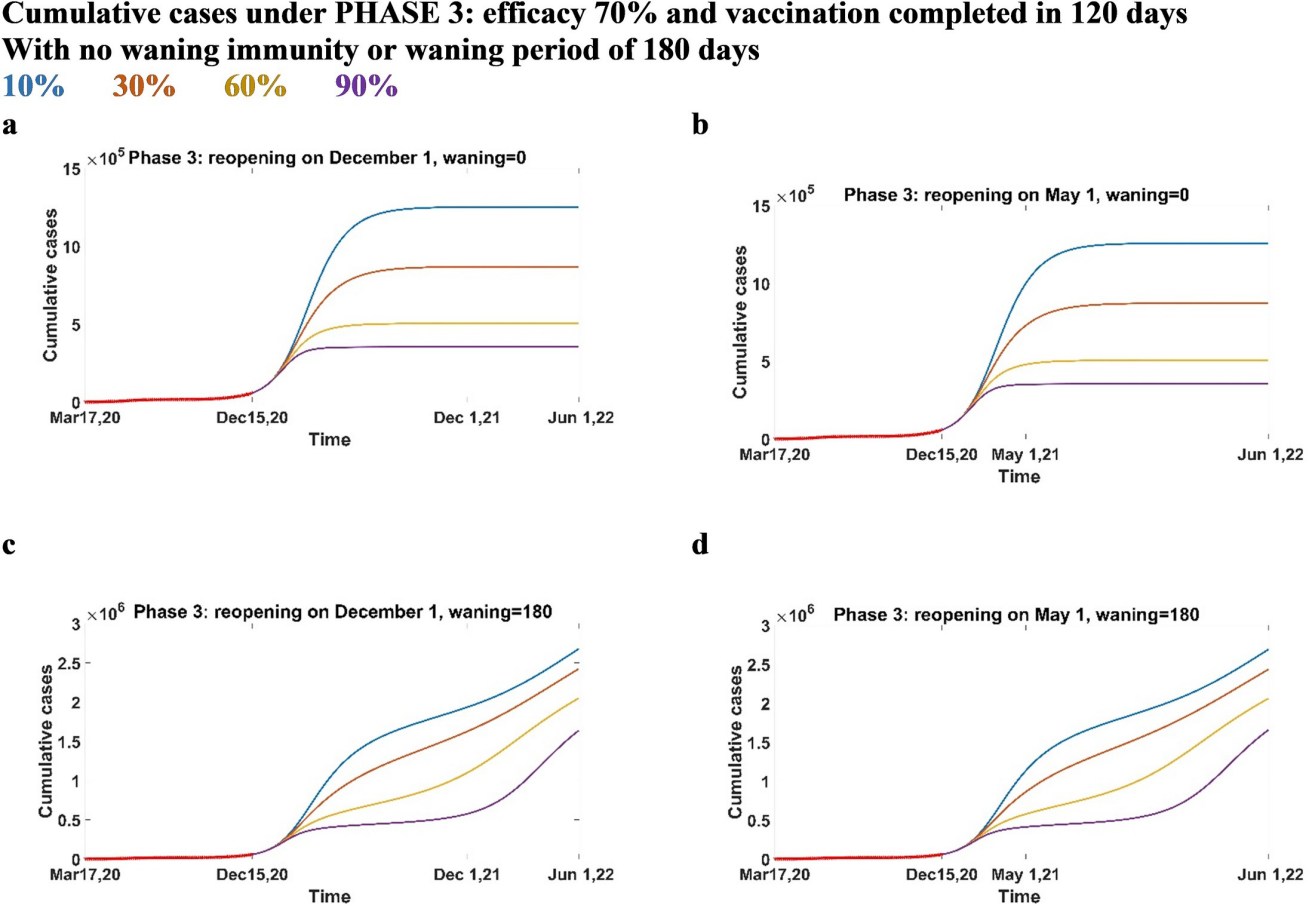
Cumulative cases under Phase 3: Efficacy 70% and vaccination completed in 120 days. Cumulative cases when the vaccine efficacy is 70%, vaccination is completed in 120 days under Phase 3 conditions, reopening in December 2021 (a-c) or May 2021 (b-d) and immunity does not wane (a-b) or wanes over 180 days (c-d).

In this section, we present predictions on cumulative cases when vaccination campaign is introduced under Phase 1 or 3 (Figs 5 and 6, respectively), while Phase 4 is reported in Supporting Information. Here, the vaccine efficacy is assumed to be 70%, and the vaccination process will take 120 days to be completed. We also assumed that there is no waning immunity, or the waning period is 180 days. In all scenarios, a lower vaccination coverage and non-lifelong immunity will give larger outbreaks if no new NPIs are enforced. With no waning immunity, the highest reported cases are under Phase 3. This is due to the fact that Phase 3 has the highest transmission among all. We observe that under Phase 1 and 3, an early reopening (Figs 5B–5D and 6B–6D) does not provide a large reduction in cases compared to a late reopening (Figs 5A–5C and 6A–6C). On the other hand, under Phase 4 (S2 Fig), reopening late is more efficient if vaccination coverage is below 30%. In all the phases, if immunity is lifelong, cases rapidly increase, reaching a plateau after few months from the beginning of vaccinations, with no resurgence. Cumulative cases show a large gap if 10% or 30% of the population gets immunized through inoculation; on the other hand, a smaller difference is evident if the coverage is 60% or 90%. The gap between the highest vaccination coverages is less visible in Phase 4 (S2 Fig). If immunity lasts only 180 days (Figs 5C, 5D, 6C and 6D), we observe that there is a resurgence of the infection with either late or early reopening. Cases (Figs 5C, 5D, 6C and 6D) increase due to the high transmission in the phase itself, but show a stabilizing trend before increasing again. If the reopening is early (Figs 5D and 6D), cases are slightly higher, and the resurgence occurs earlier than the one with a late reopening. This delayed time is more visible with highest coverages.

### Different waning periods

We also investigated other waning periods, 90 and 365 days (S3–S5 Figs). We observe that the shortest period (i.e. 90 days) is the worst-case scenario since the number of cases reported is much higher than the others. The trend is similar to the 180-day waning period, however the growth of cases is sharper. Similarly, even in this scenario, highest coverages show a plateau before the second resurgence, even if shorter than the 180-day waning scenario. If the immunity lasts one year, then the trend is similar to the life long immunity scenarios. However, with immunity waning over time and coverage less than 90%, we observe a resurgence of cases, not visible in Figs 5A, 5B, 6A and 6B.

### Different vaccine efficacy

Some of the available vaccines in Toronto have an efficacy about 90% against the virus, hence we studied the effect of a higher efficacy on cases and deaths. As expected, the highest efficacy results in reduction of cases and severe outcomes (S6–S8 Figs). If the immunity is not lifelong, we observe that the higher efficacy mitigates the exponential growth of cases, and with coverages above 60% extends the time at which the resurgence starts again. Under Phase 1 and 4, we observe that the resurgence is minimal and delayed towards mid 2022 when the coverage is 90% and late reopening.

### Different rollout

Depending on the availability of doses, the distribution of vaccine shots can be done at different rates. In our previous results, we assumed that the vaccination campaign is completed after 120 days. We also investigated a slower rollout, assuming that the total time is 360 days. If immunity is lifelong, the trend followed by the cumulative cases in S9–S11 Figs is similar to the one seen in Figs 5 and 6, S2 Fig, however the magnitude of reported infections is higher. This is due to a slower process that leaves more individuals unprotected and vulnerable to the infection. Moreover, if there is no waning immunity, we do not observe the large gap between low and high coverages, as shown in Figs 5 and 6, S2 Fig. However, if immunity wanes over 180 days, we observe that the infection spreads faster and with more cases, even for high coverages, compared to Figs 5C, 5D, 6C and 6D; this is due to the fast-waning immunity which replenishes the susceptible class sooner. Also, for coverages above 60%, the cases immediately increase and they stabilize in a plateau, until mid 2022, when we observe a slight resurgence, contrary to the shortest rollout, where the highest coverages delay the resurgence, which becomes high in a short period.

### Cumulative deaths

Cumulative deaths present similar trends noticed in all the previous scenarios and they are reported in (S12–S14 Figs).

### Predictions under Phase 2

**Fig 7:**
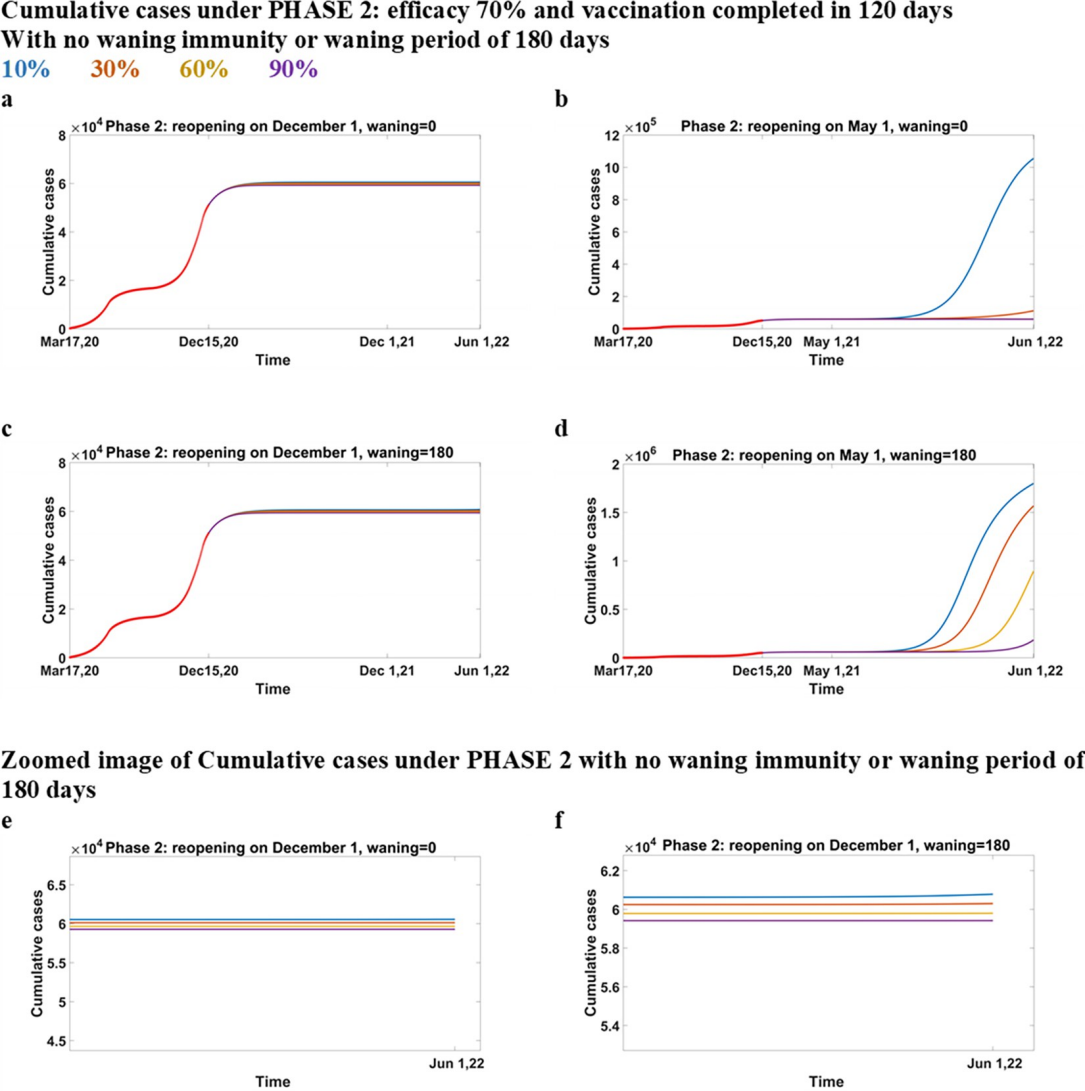
Cumulative cases under Phase 2: Efficacy 70% and vaccination completed in 120 days. Cumulative cases when the vaccine efficacy is 70%, vaccination is completed in 120 days under Phase 2 conditions, reopening in December 2021 (a-c) or May 2021 (b-d) and immunity does not wane (a-b) or wanes over 180 days (c-d). sub-figures (e-f) are zoomed images of sub-figures (a) and (c).

In this section, we present predictions on cumulative cases if vaccination is distributed under Phase 2 conditions (Fig 7). We observe that the conditions in Phase 2 provide the lowest reported cases in any reopening scenario among all the phases. A late reopening (Fig 7A–7C) shows an extremely low growth of cases over the time compared to an early reopening (Fig 7B–7D). We also observe that with no waning immunity and reopening in December, we reach a plateau, and there is no sign of resurgence by June 2022 (see zoomed figure, Fig 7E), even with low coverages. On the other hand, a slight increase is visible if the immunity waned over 180 days and only 10% of population is vaccinated (see zoomed figure, Fig 7F). If the reopening occurs in May and immunity is not waning (Fig 7B), then a large increase of cases is noticeable for 10% coverage and a smaller one for 30% coverage; however, 60% coverage appears to be enough to prevent any resurgence. On the other hand, if the reopening occurs early and the waning period is 180 days (Fig 7D), then the cases increase even if the vaccination coverage is as high as 90%.

Comparing the results under these different phases, we observe that if vaccine is given under Phase 1 or 3, the increase in cases occurs much earlier than those seen under Phase 2.

### Different waning periods

Like the previous section, we compare the results given under different waning periods. With vaccine given under Phase 2, a late reopening keeps the infection under control, even if a waning period of 90 days shows a higher increase in cases with 10% coverage compared to the other periods (S15 Fig). If the reopening occurs earlier, then we observe that fast waning (90 days) cases will increase even with high coverages; on the other hand, with immunity lasting one year, 90% coverage appears enough to prevent resurgence by June 2022.

### Different vaccine efficacy

If the vaccine efficacy is increased to 90%, then the magnitude of reported cases is smaller than the ones with 70%, but the trends remain the same for almost all coverages and reopenings (S16 Fig). With an early reopening, we observe that with lifelong immunity, 30% coverage is enough to keep the infection under control, even if a slight increase is present around June 2022. If the immunity wanes over 180 days, then 90% coverage shows a pleateau until June 2022, contrary to Fig 5D, where we observe a resurgence of cases.

### Different rollout

Like the results in Phase 1, 3 and 4, we observe that if immunity does not wane, then vaccinating people in a longer term is not efficient since cases increase. On the other hand, if immunity fades over time, then we observe that if vaccination occurs at a slower rate, slightly fewer cases are reported. This becomes more visible for higher vaccination coverages (S17 Fig).

### Cumulative deaths

Cumulative deaths follow the trend of the discussed cumulative cases. They are reported in S18 Fig.

### Sensitivity analysis

The investigation on cumulative cases and deaths when vaccination is distributed under Phase 1 conditions (S19 Fig) shows that the waning rate is positively significant, while *p*, *ρ*, *r*_*q*24_ are negatively significant. This suggests that under this scenario if the immunity wanes fast, more cases and deaths will be reported. Also, if less people decide to get tested and receive the results within 24 h and less people are vaccinated, cases and deaths increase. In this scenario, parameter related to the vaccine rollout is not significant. On the other hand, if waning immunity does not occur (S20 Fig), then sensitivity on the other parameters shows that the time at which the peak of the vaccination process becomes significant (positively), and the other parameters (*p*, *ρ*, *r*_*q*24_) remain negatively significant. Hence, in absence of waning immunity, the rollout time is important to control the infection. Sensitivity using a lower transmission scenario (Phase 2) corresponds to the one described above (S21 and S22 Figs); however, when waning immunity does not occur, the rollout time is slightly significant (PRCC slightly above 0.5).

## Discussion

We have introduced a compartmental model to examine a global vaccination strategy to control COVID-19 outbreaks given different scenarios of the vaccine’s coverage and effectiveness, and waning immunity. We considered different time points of NPI relaxation and different phases of the epidemic trajectory and testing processes. By investigating testing rates, turnaround times, and the restrictions enforced by public health, we were able to provide objective criteria to describe epidemic’s trajectories through distinct phases. We use Toronto as a case study. Our model does not directly describe an aged- structured population, but rather incorporates the activity level of the population considering different transmission and contact rates that depend on the hourly distribution, on a daily basis, of the time spent at work/school, in the community, and at home. Since activity in these settings is impacted by public health policy, this differentiation helps us understand which location drives the transmission, as measured by the reproductive number. Our data fitting shows that household transmission is much higher than transmission at work/school or community, even though the number of contacts is smaller: this can undoubtedly be attributed to the *duration* of contacts within households. Therefore, it is crucial that public health focuses on reducing transmission in the household, by promoting sanitizing and hand-hygiene processes: we suspect that, in general, less attention is paid to these measures *within* households. Other studies have also indicated household transmission as one of the main drivers of COVID-19 spread [44–46]. As expected, the introduction of vaccination reduces transmission. Moreover, to keep epidemic spread under control, increasing the number of positive tests returned within 24 hours is paramount. This finding suggests a need for increasing public health resources for the quick identification of COVID-19 positive cases.

We generated forecasts of cumulative cases and deaths following vaccine implantation, considering different levels of efficacy (70%-90%) and different time durations for waning immunity (0, 90, 180, 365 days). Vaccination is described using a piecewise function, increasing at the beginning of vaccination campaign and decreasing after reaching a peak. Our results suggest that rapid waning immunity, independently from vaccine coverage, will result in disease resurgence. Also, with higher transmissions and immunity waning over time, our results show that vaccinating 90% of the population delays the reemegence of the infection, giving public health enough time to implement new control measures. With lifelong immunity, vaccine coverage above 60% is sufficient to prevent re-emergence of the outbreak under the strictest control measures. Under epidemic conditions characterized by fewer public health restrictions and increased contact rates in the community, late relaxation of NPIs does not help in controlling the infection, unless contacts are reduced. However, in the lowest transmission scenario, corresponding to a timeframe characterized by lockdowns and low contact rates, even with a 90-day waning immunity, postponing NPI relaxation can result in decreases in cases and deaths, and control of the infection. This suggests that under the low transmission, and a large proportion of susceptibles, lifting NPIs will result in a rapid infection resurgence. Furthermore, in all scenarios, a 90% vaccine effectiveness results in further reductions in cases and deaths. Hence, we conclude that short immunity and early relaxation of NPIs are key drivers for disease resurgence. Our results confirm previous studies stating that a late reopening will result with higher cases and deaths [11, 21, 22]. Our results on the time of rollout show that with no waning immunity a faster distribution of vaccine is always beneficial. On the other hand, if immunity wanes, then giving the vaccine slowly, will delay the people who will become susceptible and so reduce the spread of the infection. Since the waning immunity process is still unknown, future work is needed to further understand its role as well as and vaccine efficacy’s.

Analysis on the sensitivity of the model’s parameters confirms the importance of testing and a fast turnaround time of tests to support vaccination strategies to reduce cases and deaths.

Our model has some limitations. Firstly, we consider the population as a whole. However, age and socioeconomic status are imperative to incorporate when vaccine policies need to be implemented. For example, in many countries that have started vaccinating, older individuals and those working in healthcare and other essential occupations have been prioritized in receiving the first doses. We are currently working on extending our work to include multiple age groups. Secondly, we assume that all symptomatic cases stay at home, following a strict self-isolation once symptoms show. However, this might not be realistic, as the compliance to self-isolation protocols seems to be variable: this aspect will be included in future work. Thirdly, we only include one vaccination dose providing full immunity, but we know that there is a gap between the doses, which is an important factor in terms of transmission.

Additionally, we assume that the hospital compartment includes all the structures where patients need extra health care, such as long-term care and hospitals, and also that deceased cases are only reported from this whole structure. However, the differentiation among all these structures needs to be included to understand better where public health needs to focus its resources. Similarly, we are ignoring internal hospital structures so that care facilities are treated as a single compartment: as some COVID-19 patients need a level of care that is treated in ICUs, a differentiation among severe cases in nosocomial needs to be included to better describe the hospitalized cases.

In conclusion, our methods, modeling the virus trajectory throughout different phases using epidemiological data combined with information on government restriction policies, make our results applicable to any geographic context for which sufficiently granular data are available. In fact, to understand the infection dynamics and the impact vaccination will have on it, it is crucial, but also sufficient, to identify the phase in the outbreak that a specific region is experiencing. Our findings suggest that public health authorities emphasize the need for the population to pay attention to the transmission *inside* households by urging strict compliance with sanitation measures upon arrival at home (wash hands, accessories, change of clothes, etc). In order to prevent COVID-19 resurgence, higher vaccination coverage is essential, along with a late relaxation of NPIs. Also, given the importance of tests’ turnaround times for the transmission, it is fundamental to reduce the time to return tests. Future work to determine the window of immunity following vaccination is needed to further refine our understanding of the required vaccine coverage to keep virus spread under control.

## Supporting information

S1 Fig *R*_0_ 4D contour plot in the *β*_*h*_, *r*_24_, *ρ* parameters space. The values of the reproduction number are presented under Phase 1 (a), 2 (b), 3 (c) and 4 (d), 0%, 30%,90% vaccine coverage, lifelong immunity and efficacy 90%.
(TIF)

S2 Fig Cumulative cases under Phase 4: Efficacy 70% and vaccination completed in 120 days with no waning immunity or waning period of 180 days. (TIF)

S3 Fig Cumulative cases under Phase 1: Efficacy 70% and vaccination completed in 120 days with waning period of 90 or 365 days. (TIF)

S4 Fig Cumulative cases under Phase 3: Efficacy 70% and vaccination completed in 120 days with waning over 90 or 365 days. (TIF)

S5 Fig Cumulative cases under Phase 4: Efficacy 70% and vaccination completed in 120 days with waning over 90 or 365 days. (TIF)

S6 Fig Cumulative cases under Phase 1: Efficacy 90% and vaccination completed in 120 days with no waning immunity or waning period of 180 days. (TIF)

S7 Fig Cumulative cases under Phase 3: Efficacy 90% and vaccination completed in 120 days with no waning immunity or waning period of 180 days. (TIF)

S8 Fig Cumulative cases under Phase 4: Efficacy 90% and vaccination completed in 120 days with no waning immunity or waning period of 180 days. (TIF)

S9 Fig Cumulative cases under Phase 1: Efficacy 70% and vaccination completed in 360 days with no waning immunity or waning period of 180 days. (TIF)

S10 Fig Cumulative cases under Phase 3: Efficacy 70% and vaccination completed in 360 days with no waning immunity or waning period of 180 days. (TIF)

S11 Fig Cumulative cases under Phase 4: Efficacy 70% and vaccination completed in 360 days with no waning immunity or waning period of 180 day. (TIF)

S12 Fig Cumulative deaths under Phase 1: Efficacy 70% and vaccination completed in 120 days with no waning immunity or waning period of 180 days. (TIF)

S13 Fig Cumulative deaths under Phase 3: Efficacy 70% and vaccination completed in 120 days with no waning immunity or waning period of 180 days. (TIF)

S14 Fig Cumulative deaths under Phase 4: Efficacy 70% and vaccination completed in 120 days with no waning immunity or waning period of 180 days. (TIF)

S15 Fig Cumulative cases under Phase 2: Efficacy 70% and vaccination completed in 120 days with waning period of 90 or 365 days. Sub-figures (e-f) are zoomed images of sub-figures (a) and (c).
(TIF)

S16 Fig Cumulative cases under Phase 2: Efficacy 90% and vaccination completed in 120 days with no waning immunity or waning period of 180 days. Sub-figures (e-f) are zoomed images of sub-figures (a) and (c).
(TIF)

S17 Fig Cumulative cases under Phase 2: Efficacy 70% and vaccination completed in 360 days with no waning immunity or waning period of 180 days. Sub-figures (e-f) are zoomed images of sub-figures (a) and (c).
(TIF)

S18 Fig Cumulative deaths under Phase 2: Efficacy 70% and vaccination completed in 120 days with no waning immunity or waning period of 180 days. Sub-figures (e-f) are zoomed images of sub-figures (a) and (c).
(TIF)

S19 Fig Sensitivity analysis on cumulative cases and deaths when vaccination is introduced under Phase 1 conditions (with waning immunity). (TIF)

S20 Fig Sensitivity analysis on cumulative cases and deaths when vaccination is introduced under Phase 1 conditions (without waning immunity). (TIF)

S21 Fig Sensitivity analysis on cumulative cases and deaths when vaccination is introduced under Phase 1 conditions (with waning immunity). (TIF)

S22 Fig Sensitivity analysis on cumulative cases and deaths when vaccination is introduced under Phase 1 conditions (without waning immunity). (TIF)

S1 Table (DOCX)
