# Supplementary figures and images for: Community structured model for vaccine strategies to control COVID19 spread: A mathematical study
Source: PLoS One. 2022 Oct 27;17(10):e0258648. doi: 10.1371/journal.pone.0258648 (PMC9612529; doi:10.1371/journal.pone.0258648)

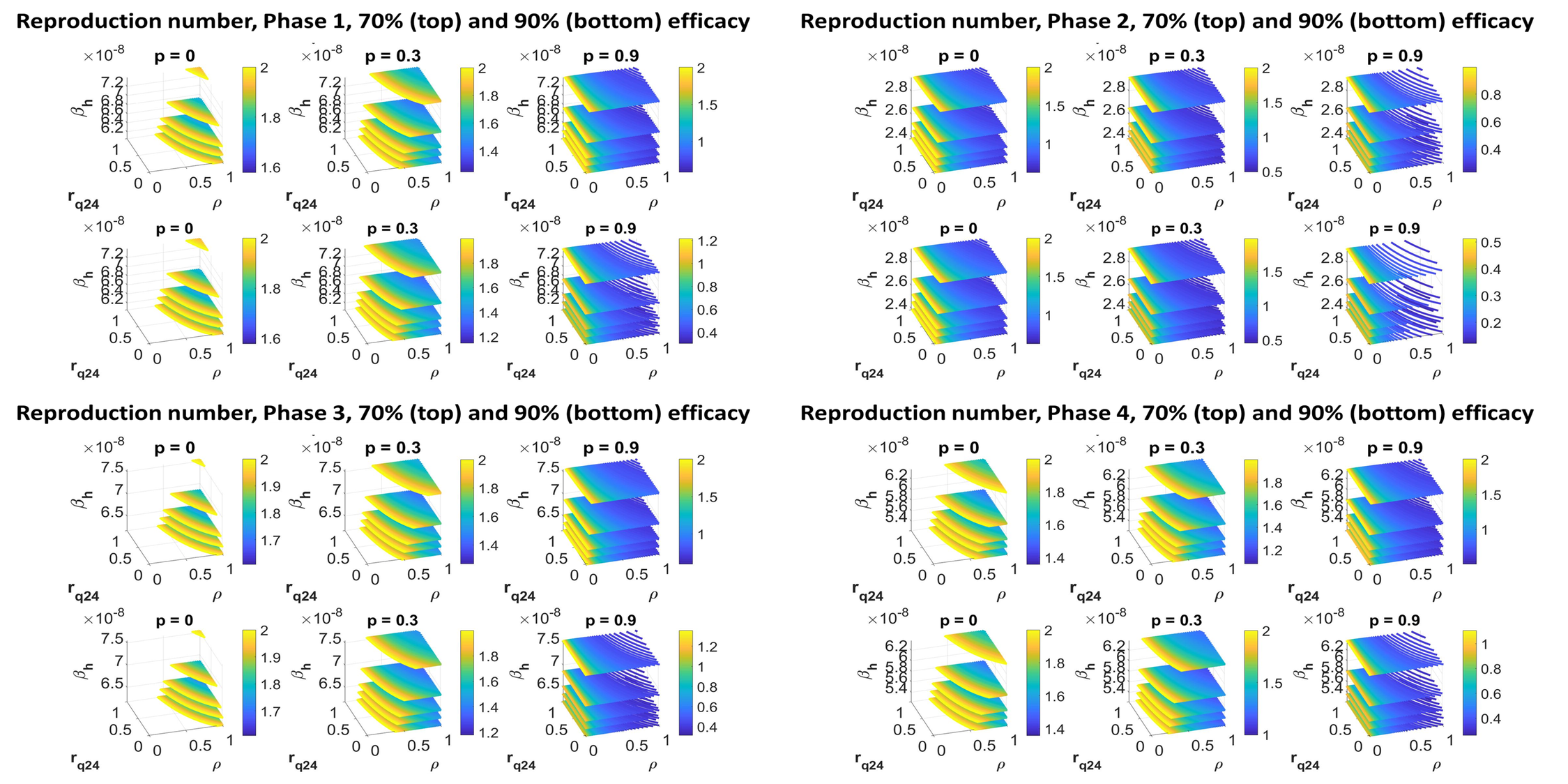

Supplement: S1 Fig — The values of the reproduction number are presented under Phase 1 (a), 2 (b), 3 (c) and 4 (d), 0%, 30%,90% vaccine coverage, lifelong immunity and efficacy 90%. (TIF) [file pone.0258648.s001.tif]

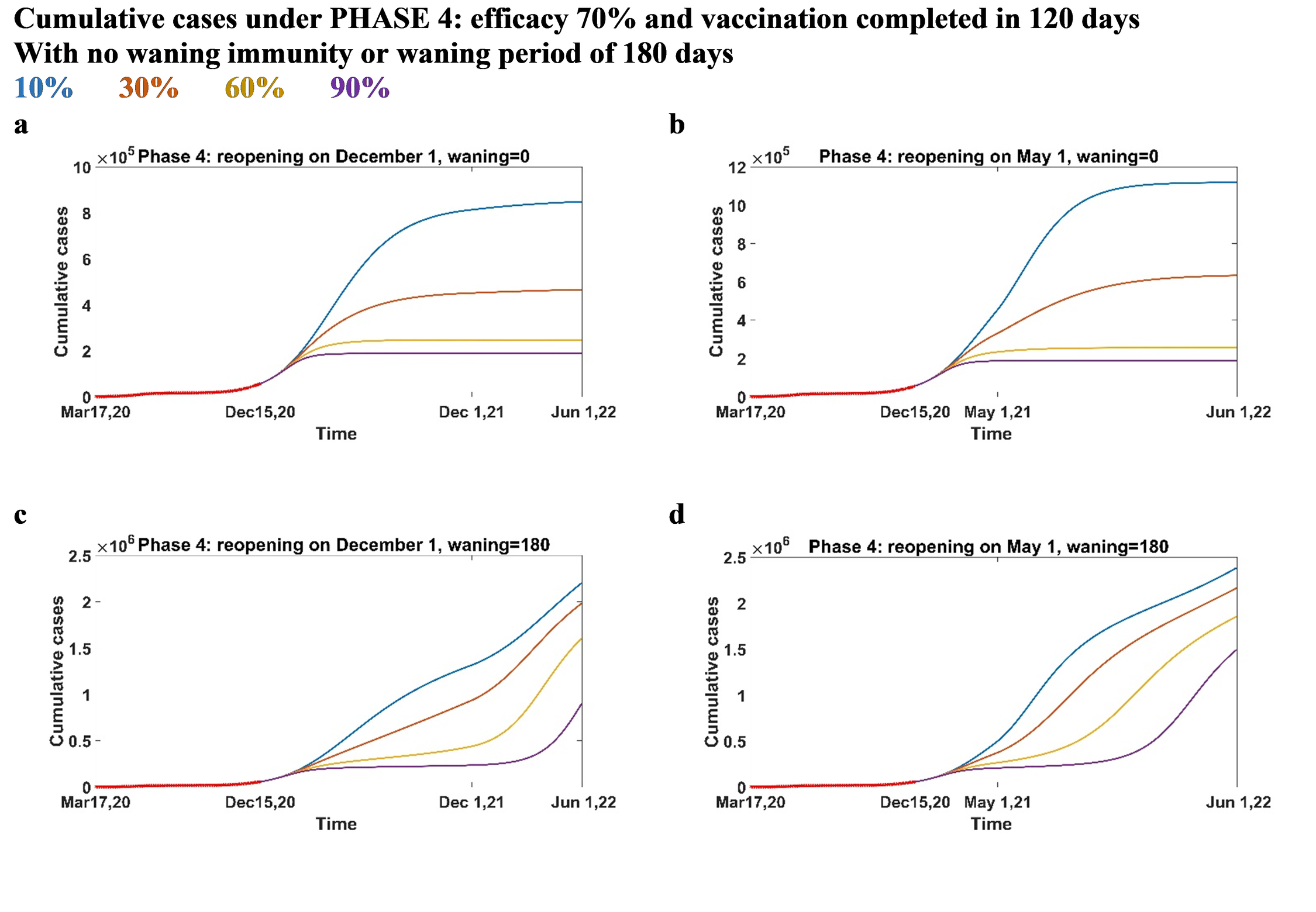

Supplement: S2 Fig — (TIF) [file pone.0258648.s002.tif]

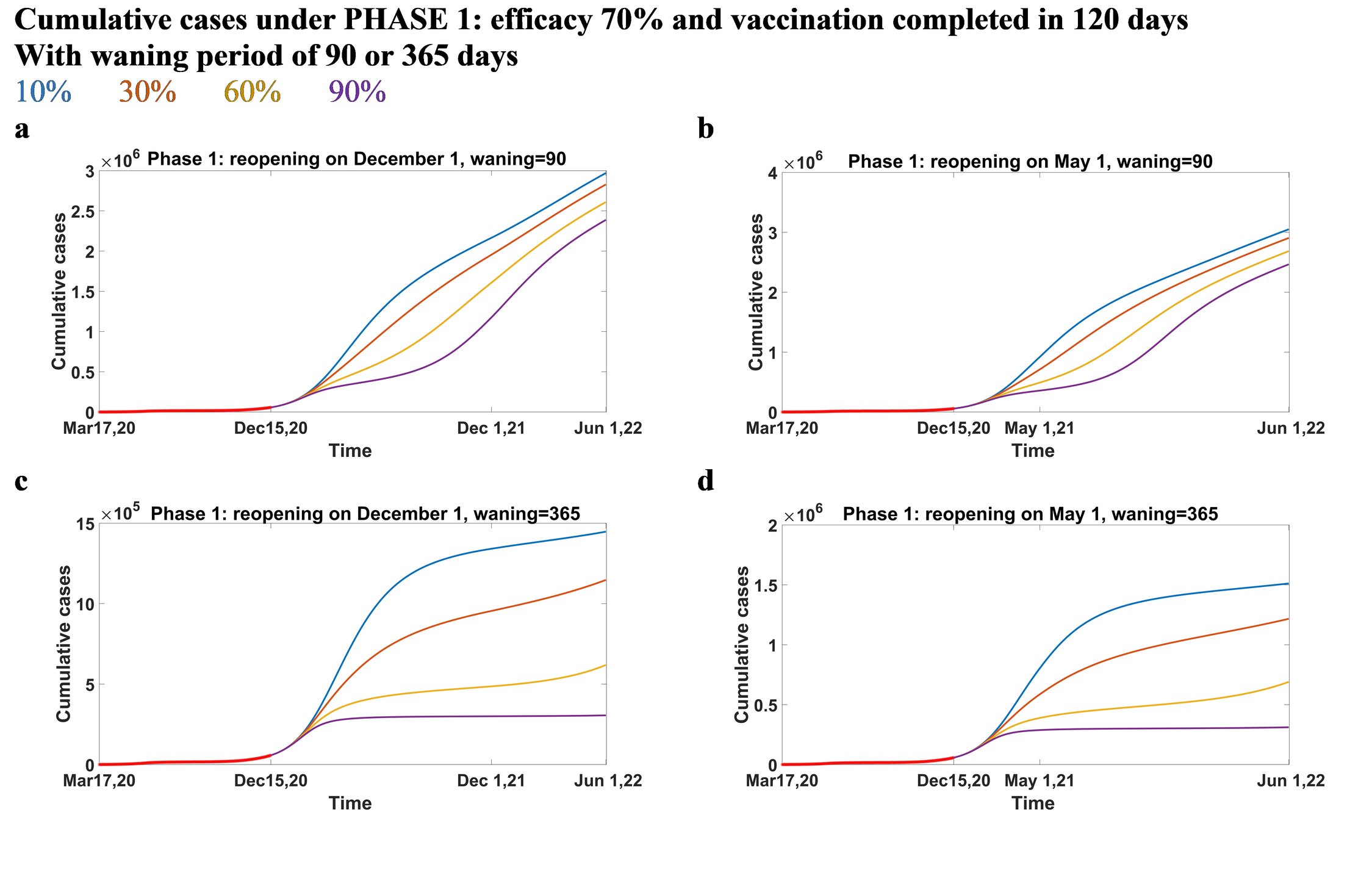

Supplement: S3 Fig — (TIF) [file pone.0258648.s003.tif]

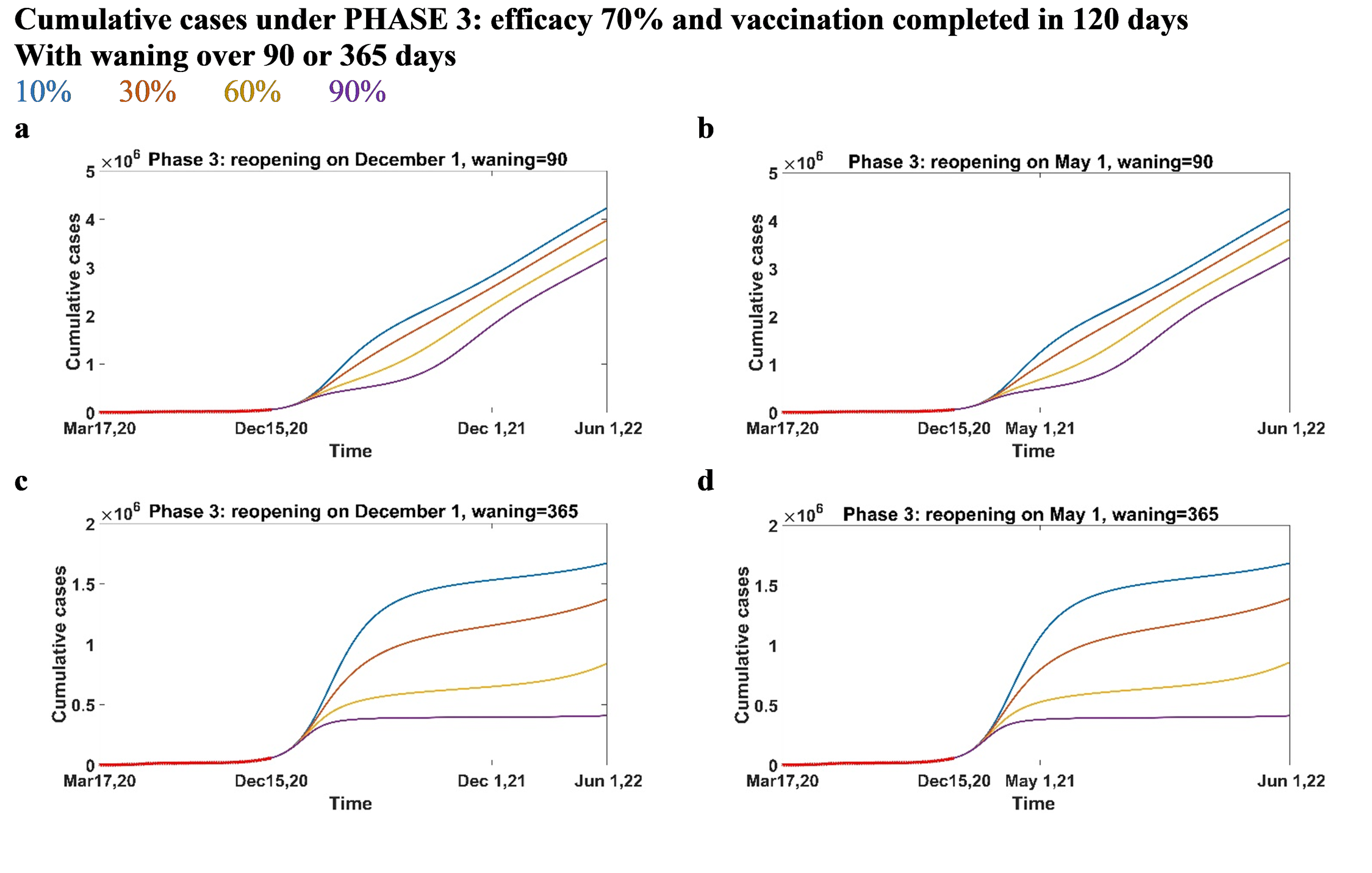

Supplement: S4 Fig — (TIF) [file pone.0258648.s004.tif]

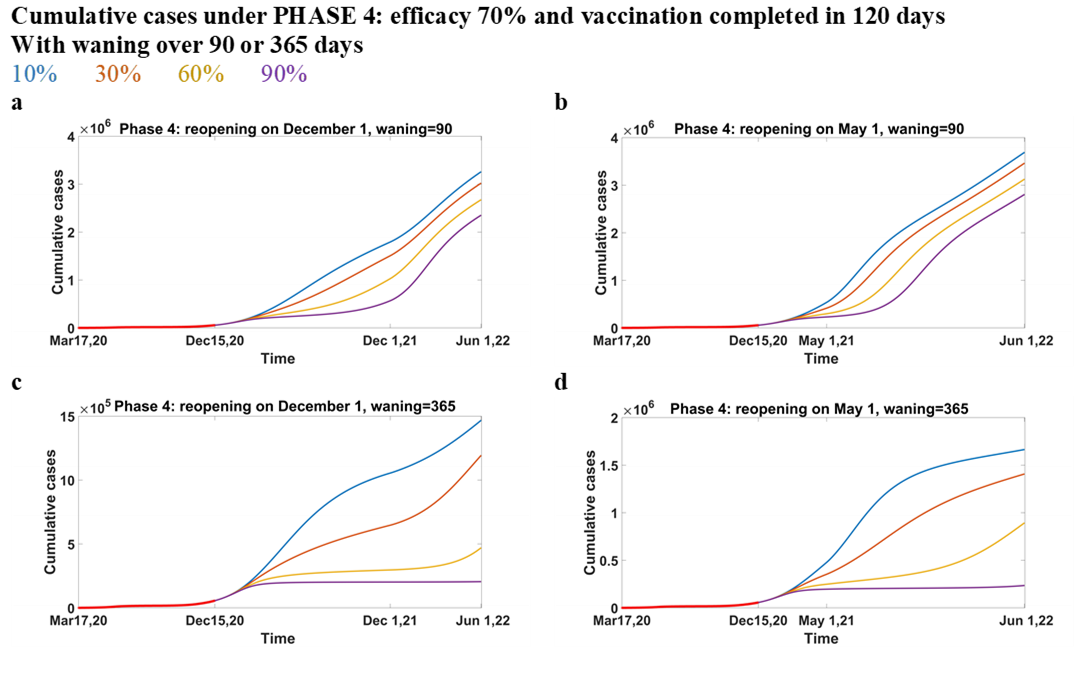

Supplement: S5 Fig — (TIF) [file pone.0258648.s005.tif]

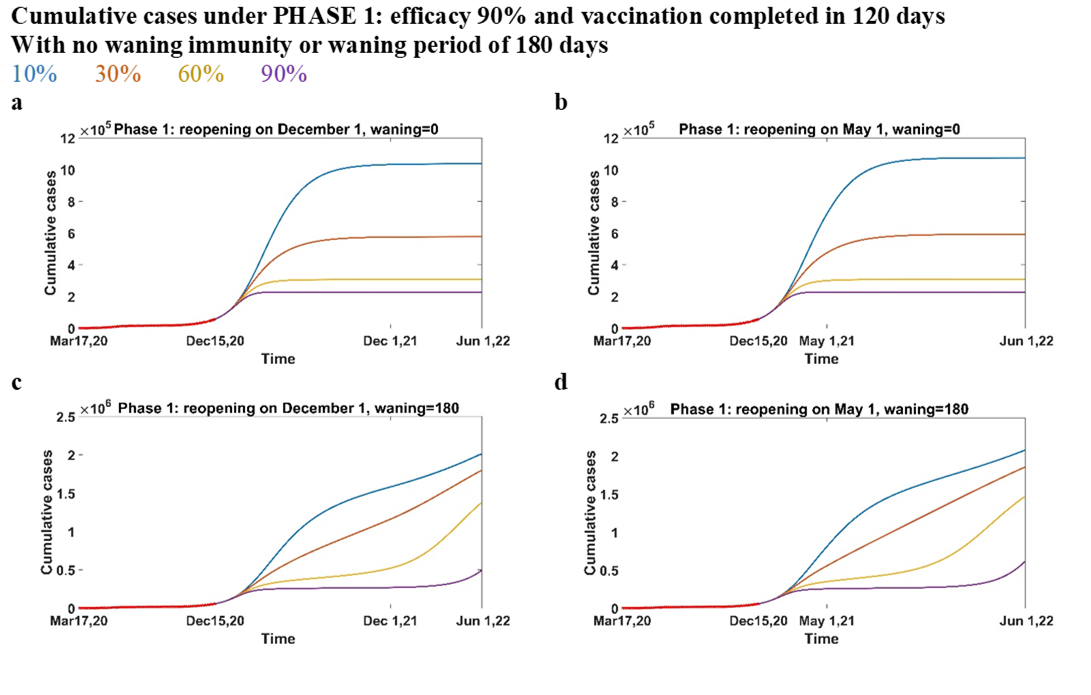

Supplement: S6 Fig — (TIF) [file pone.0258648.s006.tif]

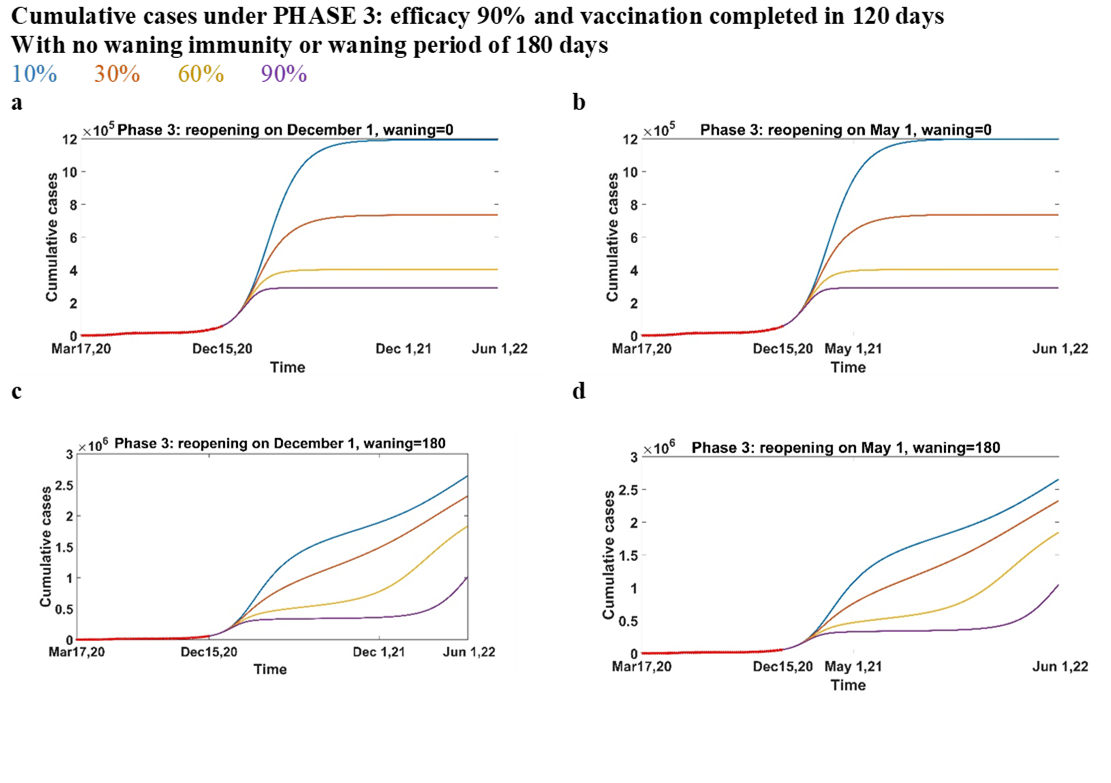

Supplement: S7 Fig — (TIF) [file pone.0258648.s007.tif]

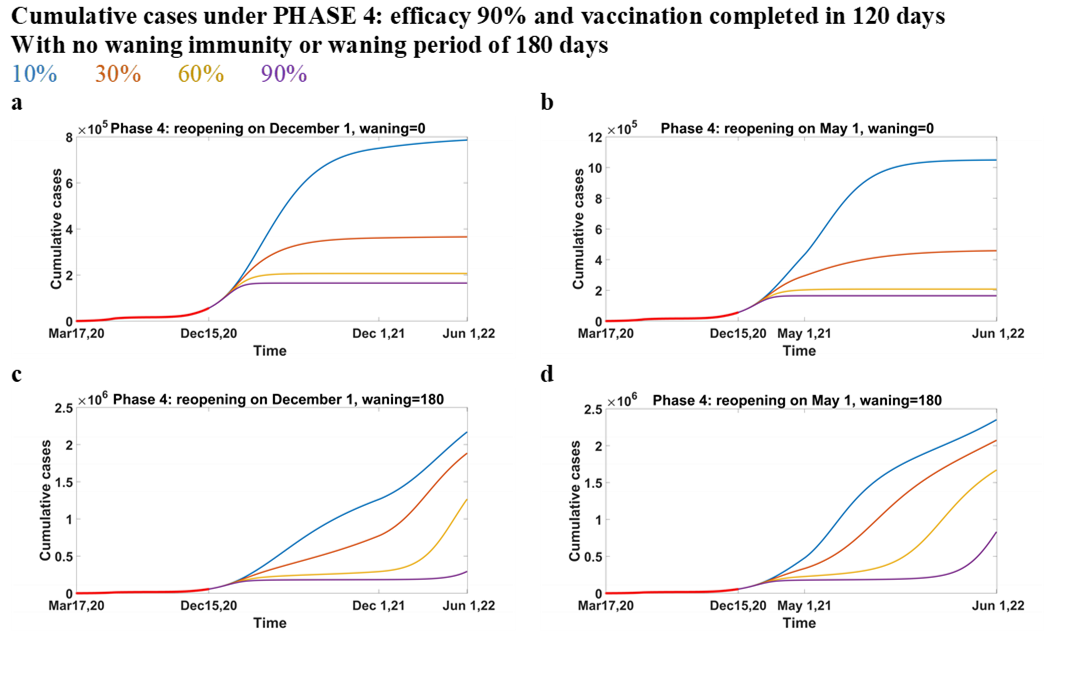

Supplement: S8 Fig — (TIF) [file pone.0258648.s008.tif]

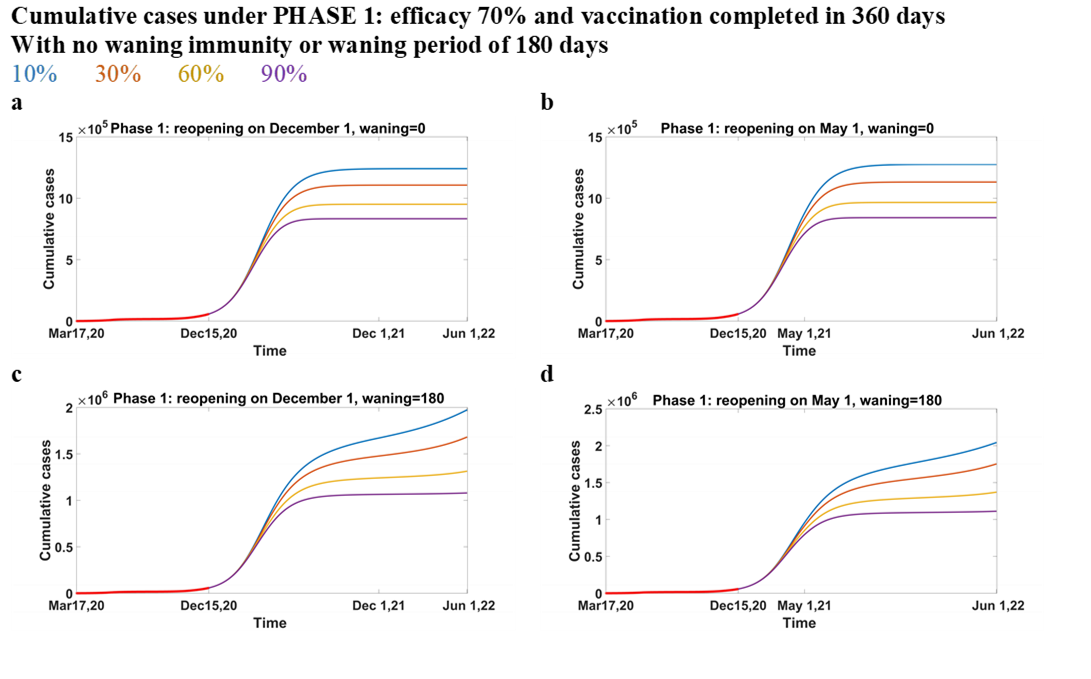

Supplement: S9 Fig — (TIF) [file pone.0258648.s009.tif]

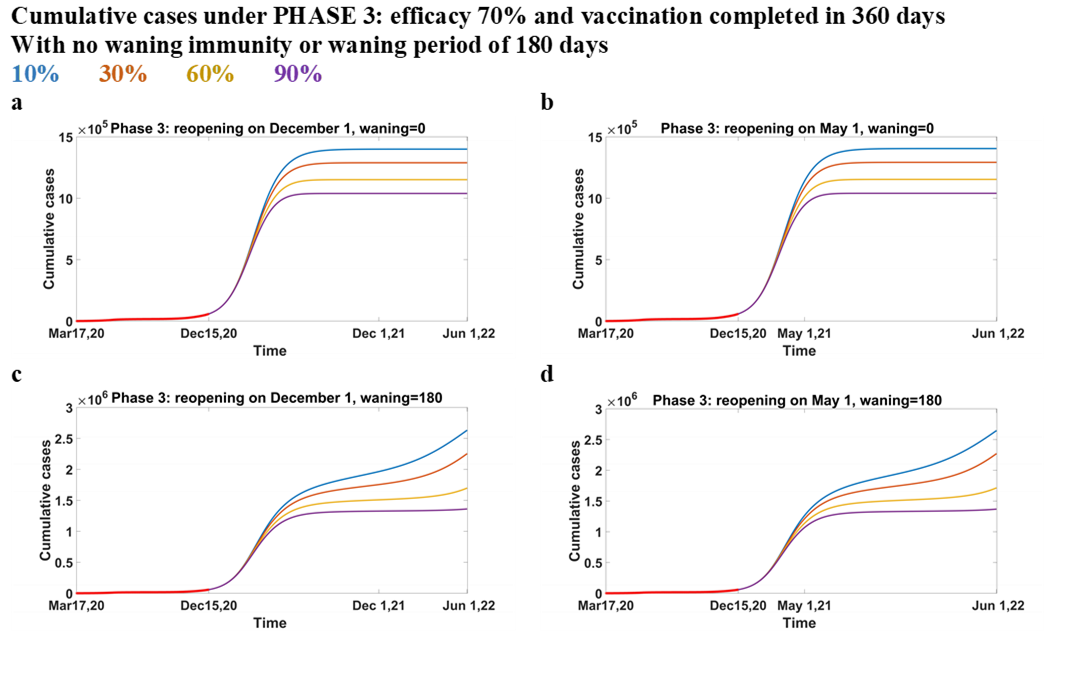

Supplement: S10 Fig — (TIF) [file pone.0258648.s010.tif]

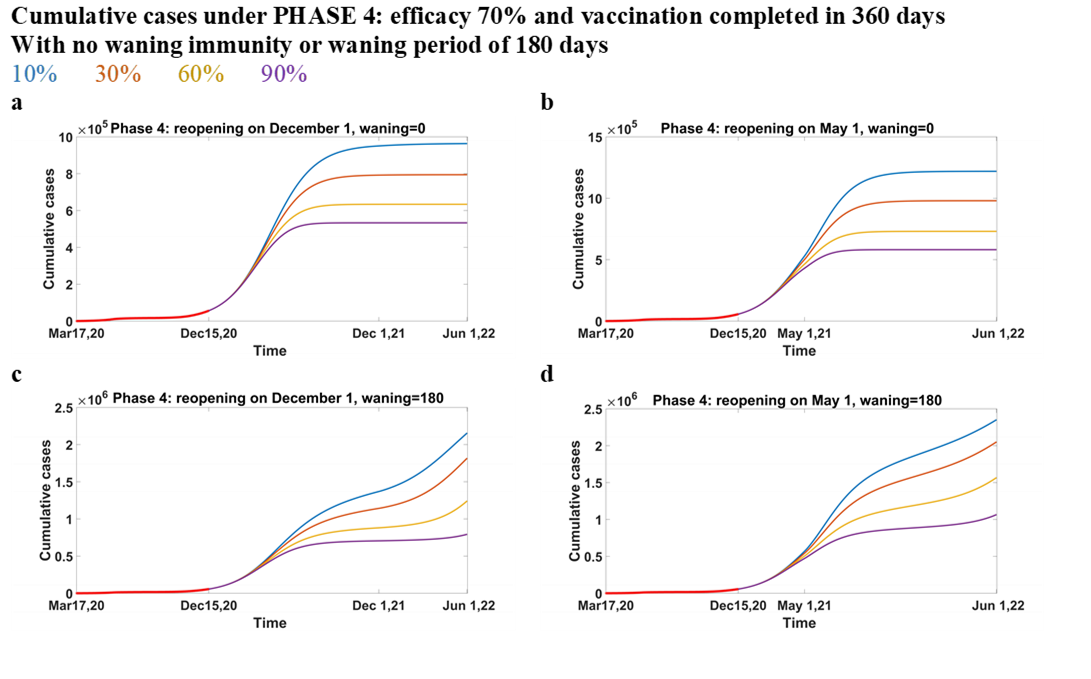

Supplement: S11 Fig — (TIF) [file pone.0258648.s011.tif]

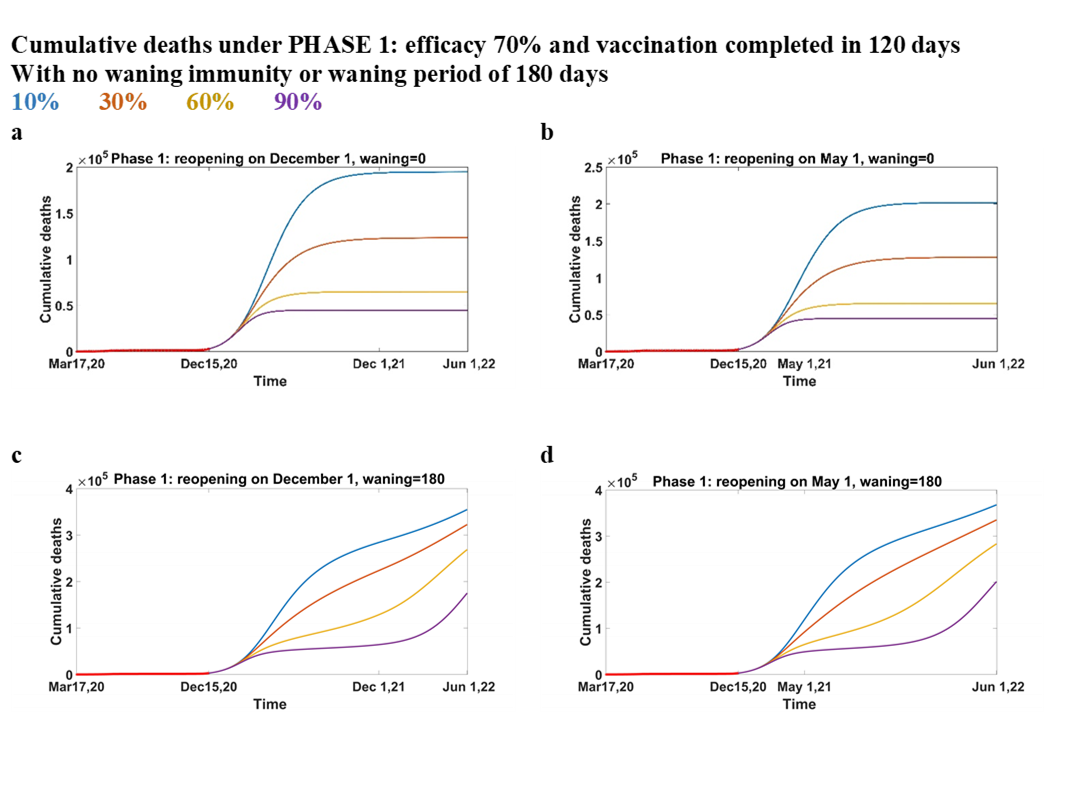

Supplement: S12 Fig — (TIF) [file pone.0258648.s012.tif]

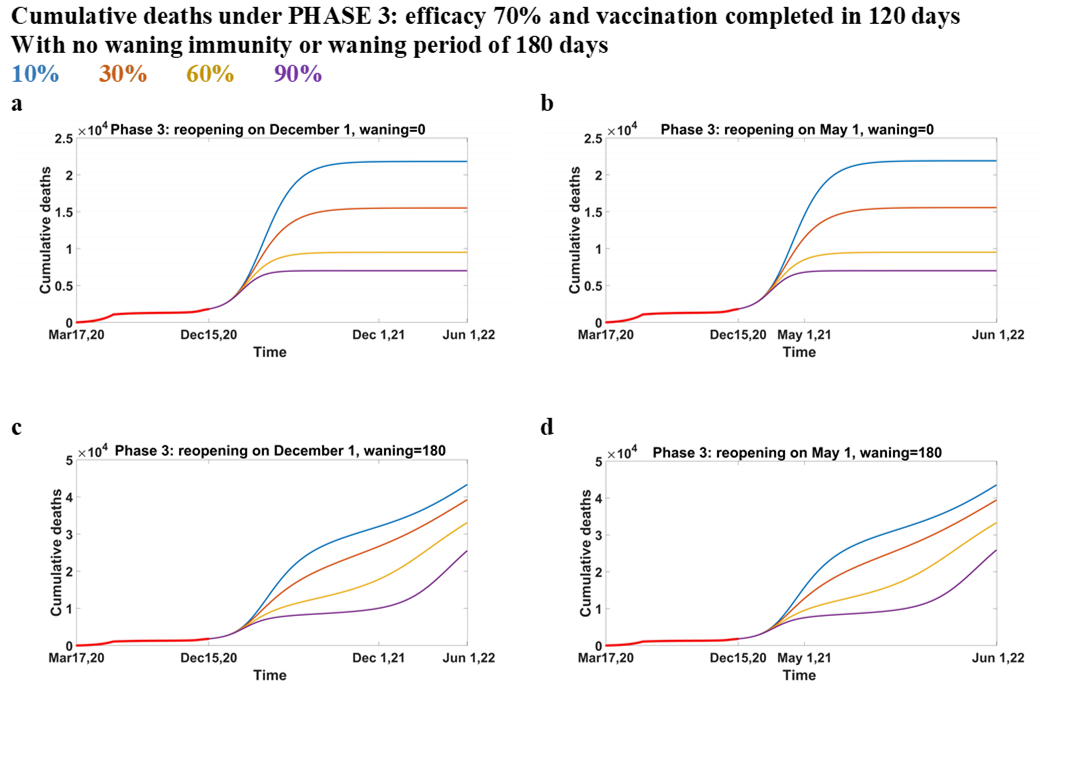

Supplement: S13 Fig — (TIF) [file pone.0258648.s013.tif]

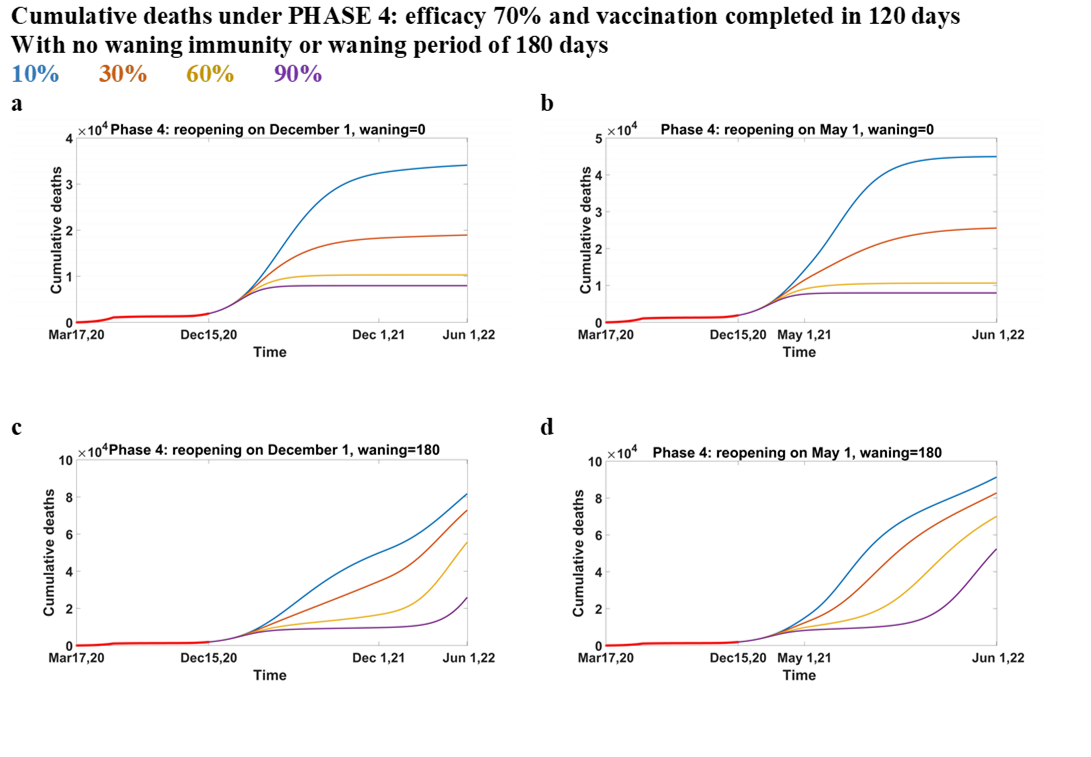

Supplement: S14 Fig — (TIF) [file pone.0258648.s014.tif]

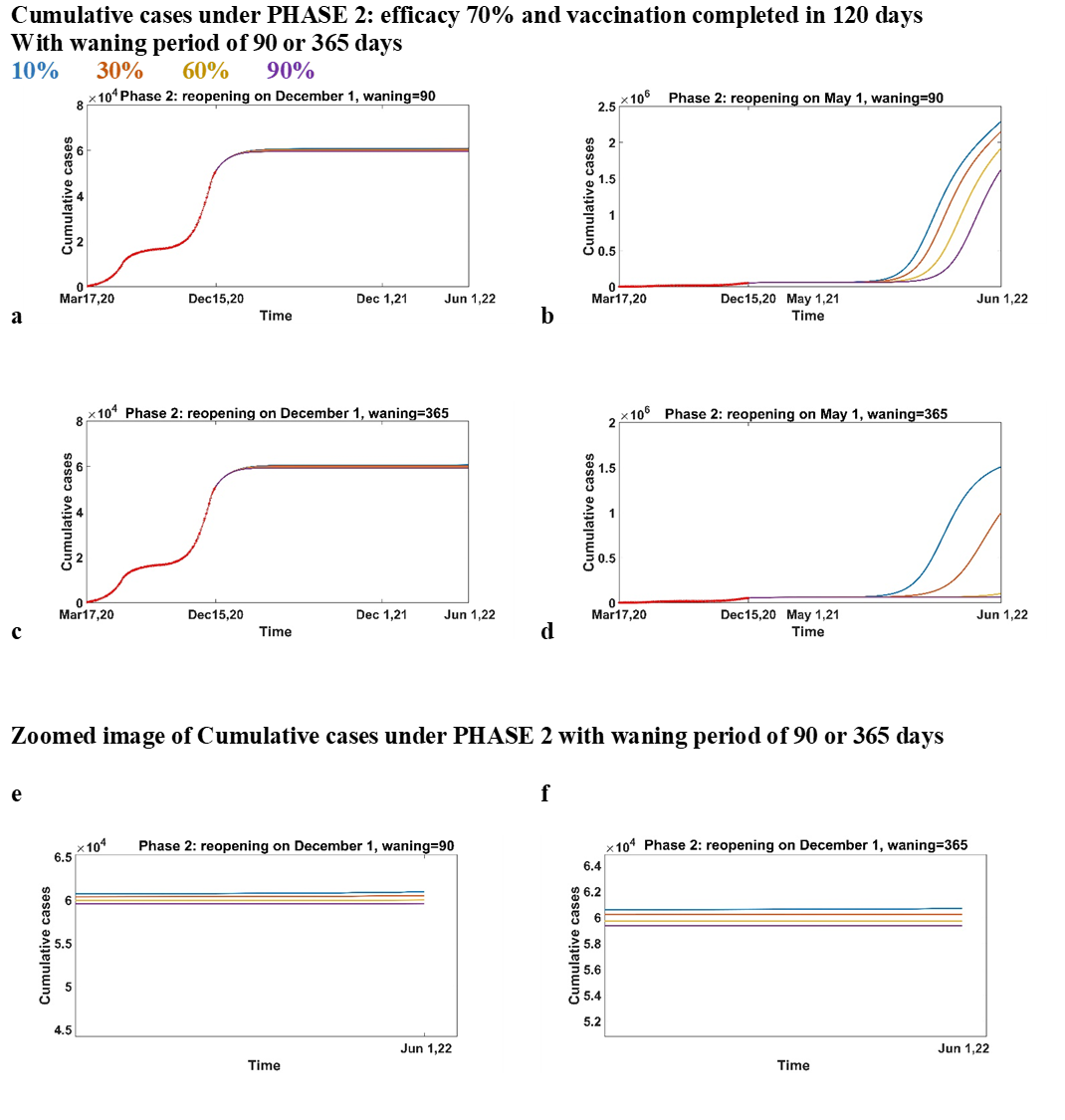

Supplement: S15 Fig — Sub-figures (e-f) are zoomed images of sub-figures (a) and (c). (TIF) [file pone.0258648.s015.tif]

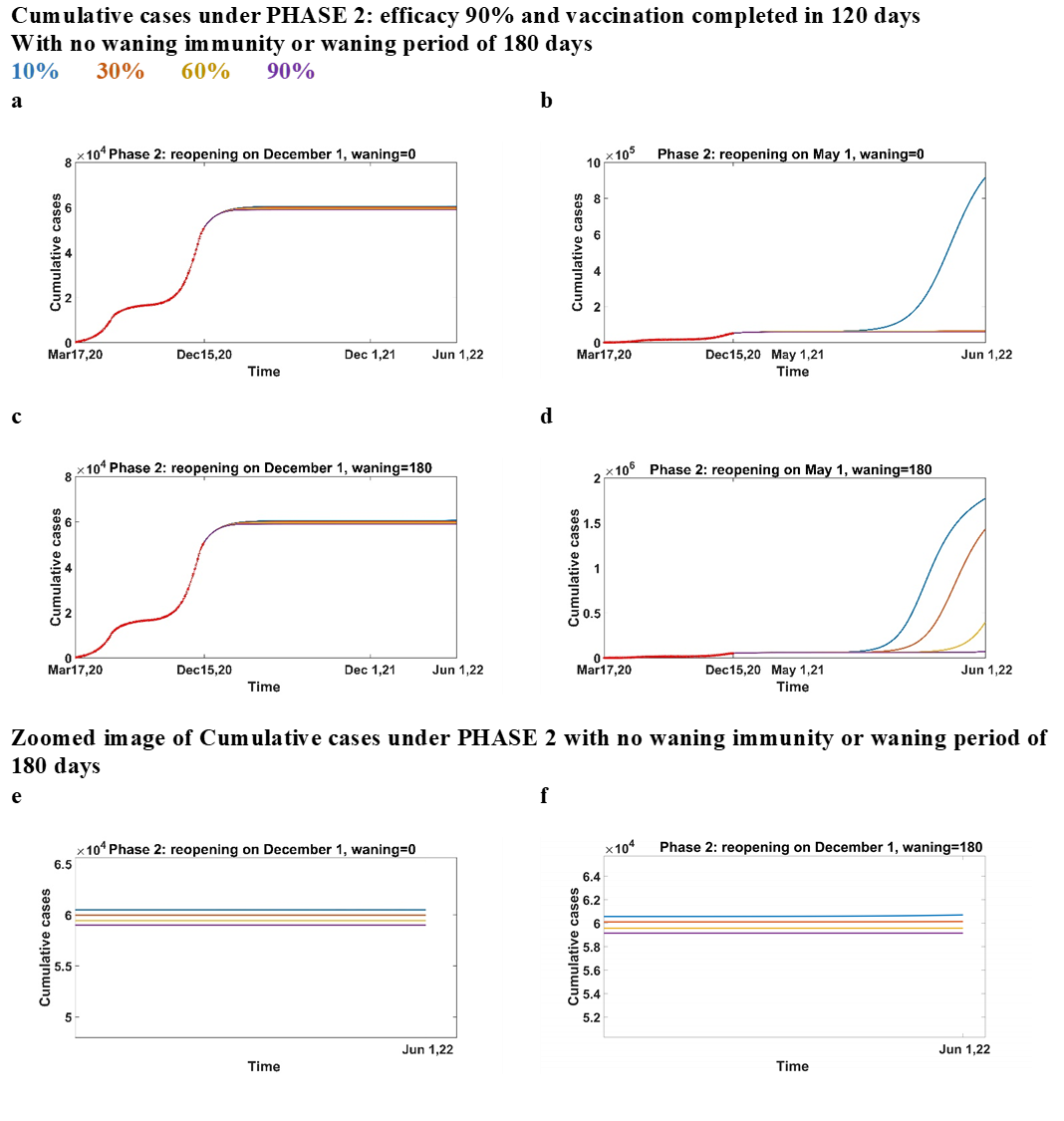

Supplement: S16 Fig — Sub-figures (e-f) are zoomed images of sub-figures (a) and (c). (TIF) [file pone.0258648.s016.tif]

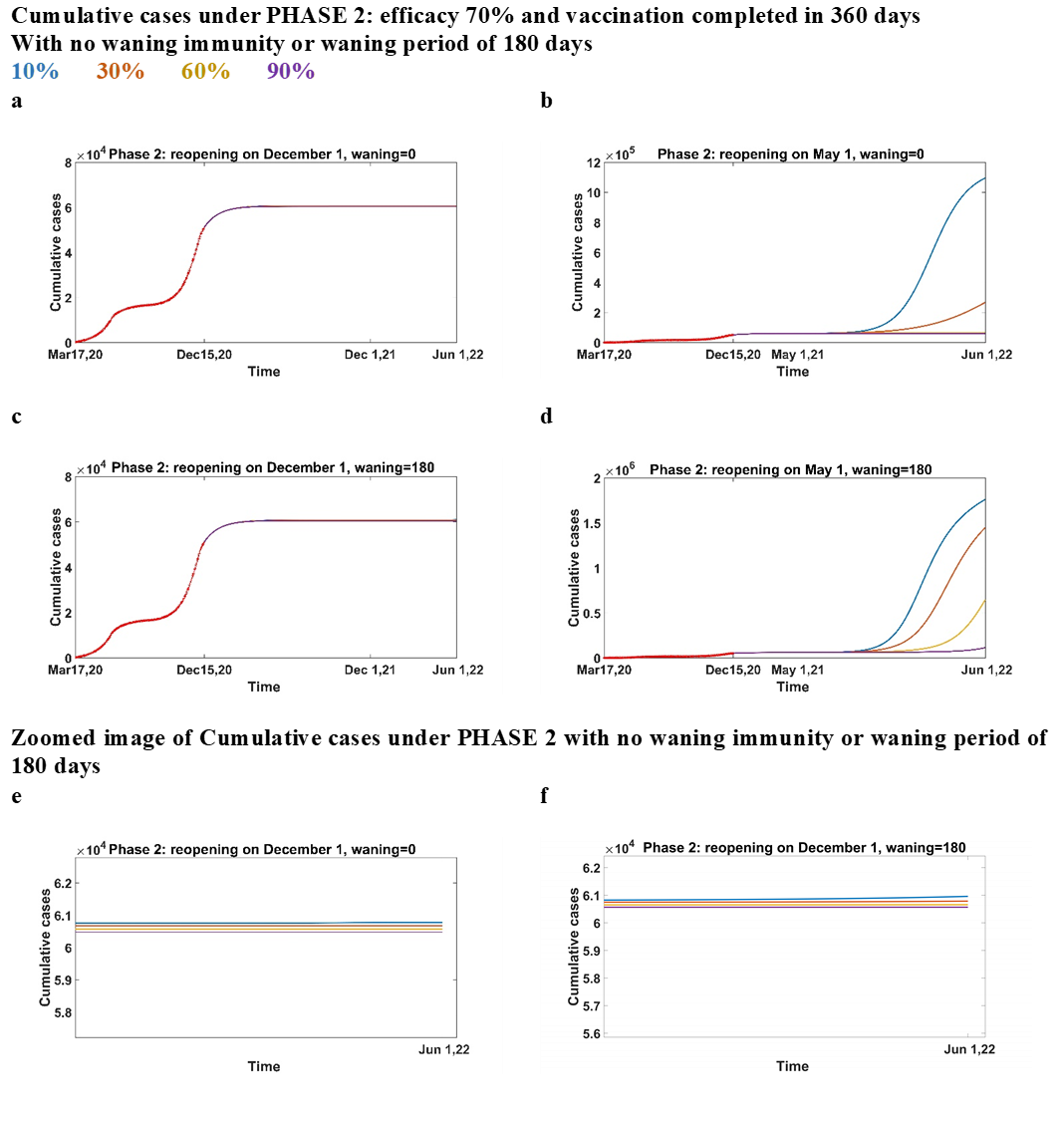

Supplement: S17 Fig — Sub-figures (e-f) are zoomed images of sub-figures (a) and (c). (TIF) [file pone.0258648.s017.tif]

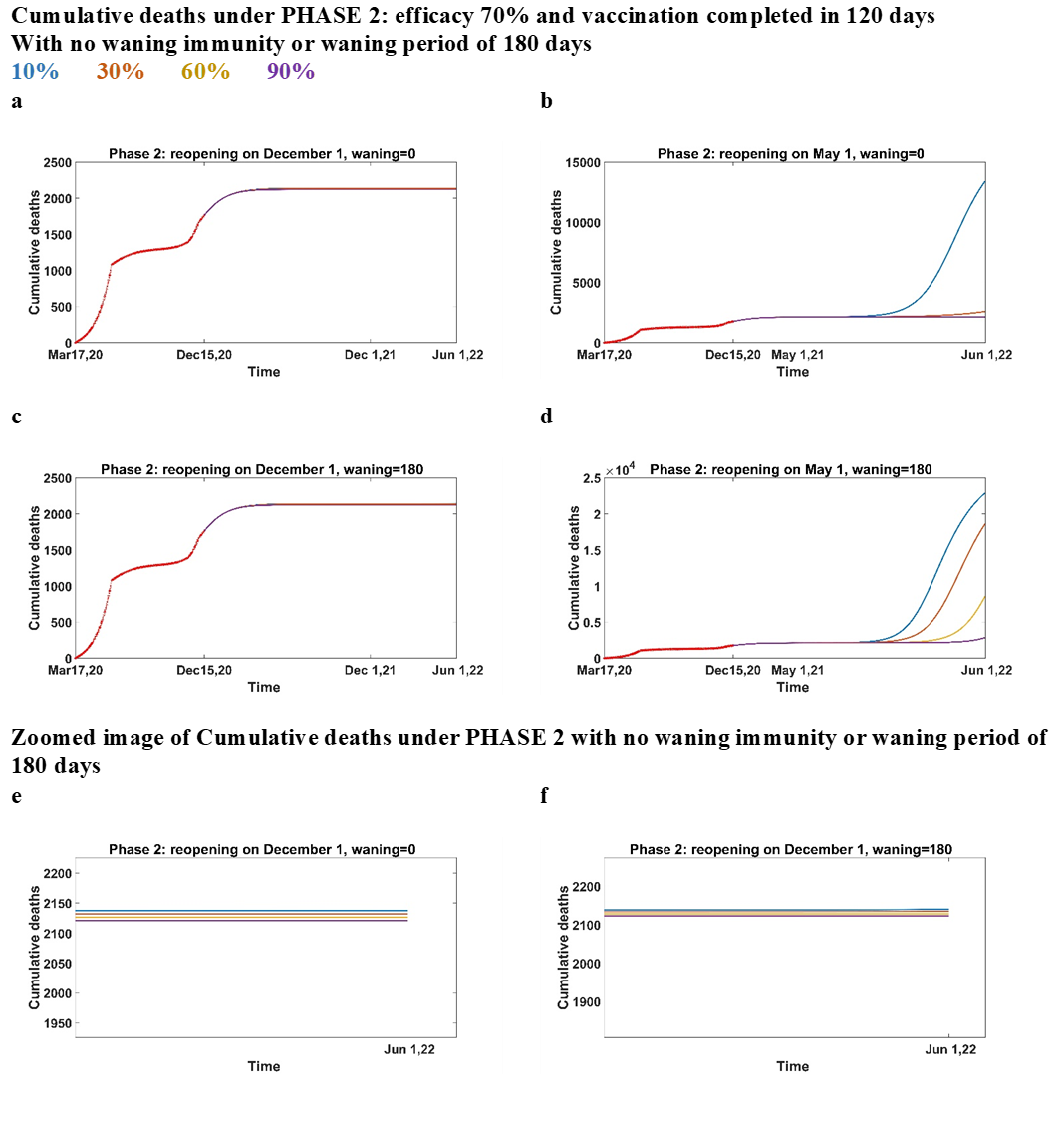

Supplement: S18 Fig — Sub-figures (e-f) are zoomed images of sub-figures (a) and (c). (TIF) [file pone.0258648.s018.tif]

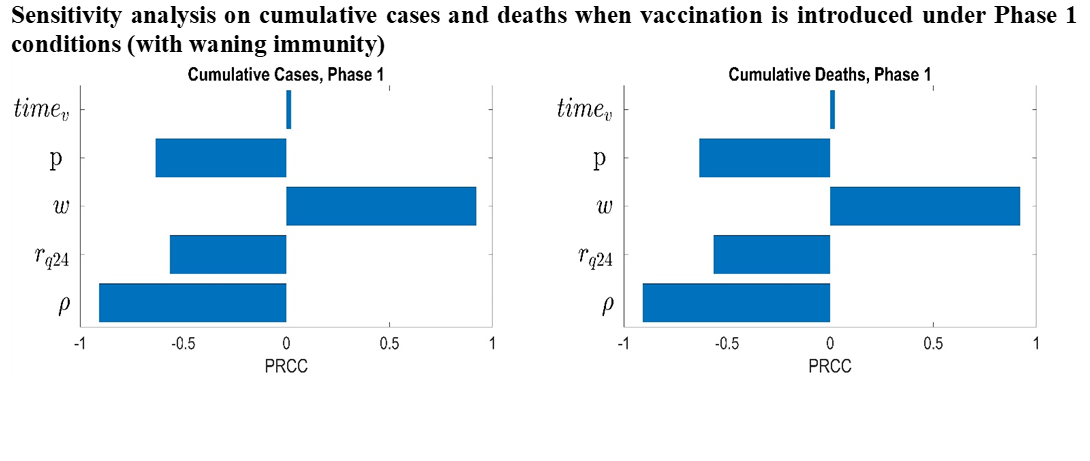

Supplement: S19 Fig — (TIF) [file pone.0258648.s019.tif]

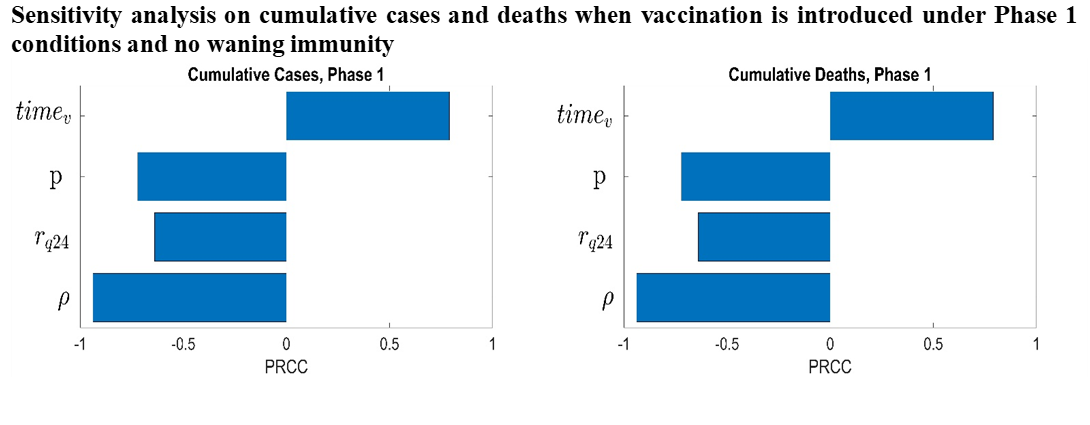

Supplement: S20 Fig — (TIF) [file pone.0258648.s020.tif]

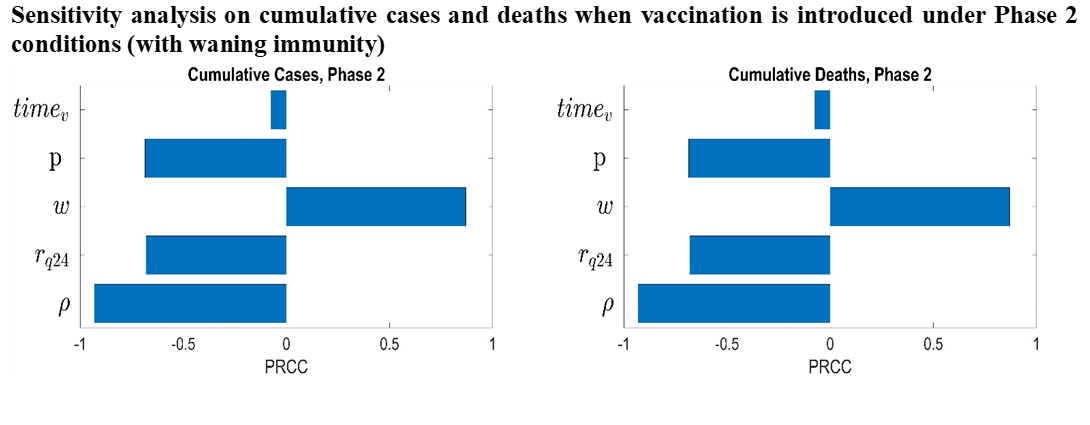

Supplement: S21 Fig — (TIF) [file pone.0258648.s021.tif]

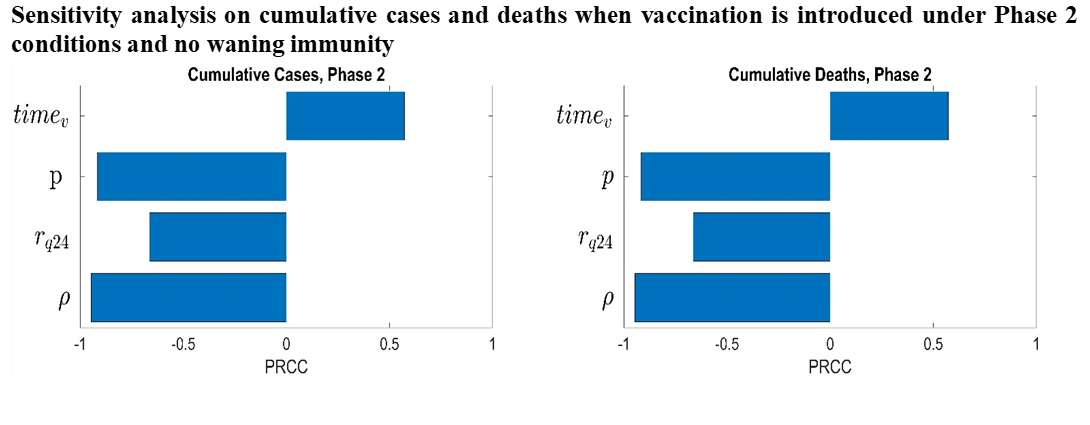

Supplement: S22 Fig — (TIF) [file pone.0258648.s022.tif]
