# Supplementary material for: Community structured model for vaccine strategies to control COVID19 spread: A mathematical study
Source: PLoS One. 2022 Oct 27;17(10):e0258648. doi: 10.1371/journal.pone.0258648 (PMC9612529; doi:10.1371/journal.pone.0258648)
Supplement: S1 Table — (DOCX) [file pone.0258648.s023.docx]

**Table S1:** Table of parameters and ranges of values used to conduct sensitivity analysis

| **Parameter** | **Definition** | **Range** |
| --- | --- | --- |
|  |  |  |
| $\boldsymbol{tim}\boldsymbol{e}_{\boldsymbol{v}}$ | Time at which the function describing the vaccination process reaches its peak | [21, 200] days |
| $\boldsymbol{p}$ | Proportion of population receiving vaccine | [0.1, 0.9] |
| $\boldsymbol{\omega}$ | Waning immunity rate | [0.0001, 1/91.25] days^-1^ |
| $\boldsymbol{r}_{\boldsymbol{q}\boldsymbol{24}}$ | Proportion of tested individuals receiving their test result within 24 hours | [0.0001, 1] |
| $\boldsymbol{\rho}$ | Proportion of infectious individuals testing | [0.0001, 1] |
